# Genome sequencing and CRISPR/Cas9 gene editing of an early flowering Mini‐Citrus (*Fortunella hindsii*)

**DOI:** 10.1111/pbi.13132

**Published:** 2019-05-21

**Authors:** Chenqiao Zhu, Xiongjie Zheng, Yue Huang, Junli Ye, Peng Chen, Chenglei Zhang, Fei Zhao, Zongzhou Xie, Siqi Zhang, Nan Wang, Hang Li, Lun Wang, Xiaomei Tang, Lijun Chai, Qiang Xu, Xiuxin Deng

**Affiliations:** ^1^ Key Laboratory of Horticultural Plant Biology (Ministry of Education) Huazhong Agricultural University Wuhan China

**Keywords:** *Fortunella hindsii*, early flowering, monoembryony, model citrus, genome sequencing, CRISPR/Cas9

## Abstract

Hongkong kumquat (*Fortunella hindsii*) is a wild citrus species characterized by dwarf plant height and early flowering. Here, we identified the monoembryonic *F. hindsii* (designated as ‘Mini‐Citrus’) for the first time and constructed its selfing lines. This germplasm constitutes an ideal model for the genetic and functional genomics studies of citrus, which have been severely hindered by the long juvenility and inherent apomixes of citrus. *F. hindsii* showed a very short juvenile period (~8 months) and stable monoembryonic phenotype under cultivation. We report the first *de novo* assembled 373.6 Mb genome sequences (Contig‐N50 2.2 Mb and Scaffold‐N50 5.2 Mb) for *F. hindsii*. In total, 32 257 protein‐coding genes were annotated, 96.9% of which had homologues in other eight Citrinae species. The phylogenomic analysis revealed a close relationship of *F. hindsii* with cultivated citrus varieties, especially with mandarin. Furthermore, the CRISPR/Cas9 system was demonstrated to be an efficient strategy to generate target mutagenesis on *F. hindsii*. The modifications of target genes in the CRISPR‐modified *F. hindsii* were predominantly 1‐bp insertions or small deletions. This genetic transformation system based on *F. hindsii* could shorten the whole process from explant to T_1_ mutant to about 15 months. Overall, due to its short juvenility, monoembryony, close genetic background to cultivated citrus and applicability of CRISPR,* F. hindsii* shows unprecedented potentials to be used as a model species for citrus research.

## Introduction

Hongkong kumquat (*Fortunella hindsii* Swingle) is a wild citrus species belonging to Citrinae group in subfamily Aurantioideae of family Rutaceae. It is widely distributed in the low‐altitude mountainous areas of South China and was initially described as ‘Shan Jin Gan’ in the ancient Chinese agronomic literature ‘Description of Citrus Fruits’ (Han, Southern Song, 1178). With a sour and spicy flavour, its fruits are cherished by southern Chinese people and are usually applied as condiment in pickle, herbal tea and Hakka chilli sauce. Besides, its dried roots and leaves could be used as a Chinese folk medicine for traumatic injury as recorded in ‘Origins of Herbal Medicines’ (Zhao, Qing, 1848). Due to its small canopy and brilliantly coloured fruits, it is also often made into miniascape for fruit‐ornamental and gardening purposes. On the other hand, previous studies have highlighted its unique features such as the smallest hesperidium among citrus species (Swingle, 1967), and very early and continuous flowering (Ye, 1985). Therefore, *F. hindsii* has always been considered as a potential model species for citrus research. For example, it is a genotype with the highest rate of callus induction among all citrus species, and callus can even be induced from its seeds or roots (Deng and Zhang, 1988). The transgenic system of *F. hindsii* has been established and applied for gene function studies involved in carotenoid metabolism (Cao *et al*., 2015; Zhang *et al*., 2009). However, all previously used *F. hindsii* accessions were derived from polyembryonic seeds (adventitious nucellar embryony) like most citrus varieties. The superiority of nucellar embryo in germination process would consequentially lead to the result that the sown seedlings are essentially clones of their maternal parent (Wang *et al*., 2017). The property of asexual reproduction will cause the high dependence of the gene function research of citrus on the first generation of transgenic plants (T_0_). Therefore, further and more accurate functional explorations of candidate genes are always hindered by the lack of the second transgenic generation (T_1_) with homozygous insertion of the candidate genes. Hence, to explore desired genotypes o*f F. hindsii* (with sexual reproduction) more suitable for gene function and genetic studies, a germplasm survey on wild Hongkong kumquat along the mountains in the southern China had been carried out during 2009–2015 by our group. As a result, nine monoembryonic and sexually reproductive *F. hindsii* individuals were originally identified in Fujian province (Chen, 2011; Zhang, 2013), which were designated as ‘Mini‐Citrus’.

Citrus is one of the most widely cultivated and economically important fruit crops (14.4 million Ha of production area and 140.3 million tonnes of yield worldwide, 2016, FAO statistics, http://www.fao.org/faostat/en/). However, both genetic and ‐omics studies of citrus are confronted with great challenges due to its unique characteristics. As mentioned above, most cultivated citrus varieties asexually reproduce polyembryonic seeds, with the exception of pummelo, citron and ichang papeda. Thus, forward genetic methods that depend on recombination and segregation are not applicable for most citrus varieties theoretically. Due to the characteristic of asexual reproduction of citrus, the purposive construction of genetic populations whether by crossing or selfing has been extremely limited. Although embryo‐rescue technique could be a solution to overcome this obstacle (Aleza *et al*., 2010a), it is complicated and time‐consuming. Secondly, the juvenile period of citrus species is as long as about 5–10 years (Chen and Hu, 1986; Krajewski and Rabe, 1995), making it slow and almost infeasible to identify the interested phenotypes of the fruit by whether forward or reverse genetic approaches. Thirdly, most citrus genomes are highly heterozygous (Shimizu *et al*., 2017; Xu *et al*., 2013), which poses great challenges to the *de novo* assembly of diploid genomes and negatively impacts the reliability and integrity of the final genomes (Jaillon *et al*., 2007; Potato Genome Sequencing *et al*., 2011). The high heterozygosity together with the polyembryony and long juvenility indeed makes it impractical to breed highly homozygous citrus germplasms via selfing like in herbaceous annuals (Badouin *et al*., 2017) or even some other woody perennials (Jaillon *et al*., 2007). Therefore, the previously published high‐quality citrus genomes were generally relied on occasional haploids obtained from gynogenesis during long‐term tissue culture (Wang *et al*., 2017; Wu *et al*., 2014).

As plant research has entered the post‐genomic era, CRISPR/Cas9 system has emerged as an efficient tool for gene function studies and is widely applied in the research of model plants and crops (Liu *et al*., 2015). However, few successful applications of CRISPR/Cas9 have been reported in citrus (Jia *et al*., 2017; Peng *et al*., 2017; Zhang *et al*., 2017), and the current model plant systems, such as *Arabidopsis thaliana* and tomato, have rather limited applications in citrus functional genomics due to the asynchronous development patterns, distinct organs and highly heterogenous genetic backgrounds (Guo *et al*., 2012; Liu *et al*., 2016; Lu *et al*., 2016; Zeng *et al*., 2013). In addition, gene function studies of citrus are also inherently restricted by the unstable and complicated chimera (having both transgenic and escaped cells) of T_0_ transgenic lines, which is formed during the regeneration process (Jia *et al*., 2017; Zhang *et al*., 2017). Meanwhile, a previous report has highlighted that in CRISPR/Cas9 T_1_ transgenic plants, the targeting mutations would be uniform (biallelic, heterozygous, homozygous and chimeric) mutations (Ma *et al*., 2015a). However, a more appropriate T_1_ citrus plant is also limited by the long juvenility and asexual reproduction (polyembryony) features of citrus species. Thus, it can be expected that a CRISPR system applicable in monoembryonic *F. hindsii* with short juvenility will further facilitate the function analysis of important genes in citrus and pave the way for further experimental utilization of this valuable germplasm.

In the present study, with the aim to exploit *F. hindsii* as the ‘model citrus’, we performed *de novo* sequencing and assembly of its genome as well as a global gene expression analysis across *F. hindsii* life cycle. The growth and phenological features of *F. hindsii* under cultivation conditions were systematically examined and presented for the first time. Moreover, the CRISPR/Cas9‐mediated targeted mutagenesis system was validated to be applicable in *F. hindsii* for the first time and its efficiency was elucidated.

## Results

### Characterization of *F. hindsii* with Short Juvenility and Monoembryony

**Figure 1:**
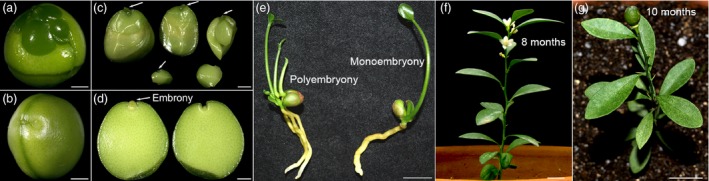
Characterization of *F. hindsii* with Monoembryonic Seed and Short Juvenility. (a) Seed of polyembryonic *F. hindsii* (without episperm). (b) Seed of monoembryonic *F. hindsii* (without episperm). (c) Disassembled seed of polyembryonic *F. hindsii*. (d) Disassembled seed of monoembryonic *F. hindsii*. (e) Germination morphology of polyembryonic and monoembryonic seeds of *F. hindsii*. (f) Flowering of 8‐month‐old *F. hindsii*. Bar = 1 cm. (g) Fruit‐bearing of 10‐month‐old *F. hindsii*. Bar = 1 cm.

During 2009 to 2015, a total of 838 *F. hindsii* wild samples and landraces were collected from 53 locations of Fujian, Jiangxi, Zhejiang, Guangdong and Hunan provinces. Among them, 25 individuals identified at Longyan, Fujian produced monoembryonic seeds (Figure 1a–e and Table S1), which were designated as ‘Mini‐Citrus’. To fix the monoembryony feature and breed highly homozygous accessions for experimental utilization and genome sequencing, successive selfing was conducted on the initially found nine Mini‐Citrus accessions (S_0_ generation, with 55.60%–74.10% of homozygosity estimated by SSR markers, Table S2). A total of 30 S_1_, 118 S_2_ and 28 S_3_ accessions were generated with average homozygosity 77.8%, 84.6% and 90.74%, respectively (Table S2). Among them, six outstanding S2 and S3 accessions showed very low genome‐level single nucleotide heterozygosity (0.62%–1.07%; Table S3 and Figure S1). The S3 accession ‘S3y‐45’ (Figure S2) showed the lowest genome heterozygosity (0.62%) which can be defined as homozygous line and is favourable for genome sequencing and assembly; the genome size of *F. hindsii* was estimated to be ~389 Mb with a repeat ratio of ~14.3% (Table S4).

To understand the botanical features of *F. hindsii* for further utilization, cultivation experiment was conducted with population S_1_. Under cultivation conditions, 70% of *F. hindsii* seedlings blossomed during their first year with a juvenile period of about 8 months (after seed sowing; Figure 1f–g and Table 1), which is the shortest juvenility so far among Citrinae species. The overall percentage of monoembryonic seeds was ~90%, indicating the stable monoembryonic phenotype of *F. hindsii* (Table 1). The 1‐year‐old seedling showed a very small canopy (6.29 × 5.12 cm) and low height (15.29 cm), indicating its potential of indoor cultivation (Table 1). During the first anthesis, each seedling developed 1.81 flower buds and bore 1.33 fruits on average, with each fruit containing an average of 1.27 seeds. In the second year, all the accessions blossomed, with each tree developing 6.00 flowers and bearing 3.88 fruits and 1.38 seeds in each fruit averagely. Three‐year‐old tree developed 19.00 flowers and bore 11.60 fruits, with each fruit containing 1.70 seeds on average. The adult *F. hindsii* exhibited continuous flowering from June to October with 3–5 rounds of flowering per year (at Wuhan, E114.37, N30.48). The flower buds of *F. hindsii* were always formed on the apical shoot or subapical axillary during the first year, but mainly developed on leaf axillary for older trees (Figure S3). The fruitlets could simultaneously develop with flower buds, and the seeds would mature till fruit colour‐breaking. The mature fruit was hesperidium with a nearly global shape in an average diameter of about 1 cm. The colour of mature fruits was yellow or orange, and would turn to reddish orange or even crimson in late winter.

**Table 1:** The botanical characteristics of *F. hindsii*

Characteristics	First year	Second year	Third year
Juvenile period (days)	248.31 ± 22.82	576.83 ± 15.34	NA
Blossom rate	70 ± 5%	100%	100%
Height (cm)	15.29 ± 2.22	25.76 ± 4.89	35.99 ± 5.47
Canopy (cm)	6.29 × 5.12	25.00 × 18.96	36.75 × 28.57
Leaf number	15.10 ± 2.36	34.98 ± 11.53	90.60 ± 32.53
Leaf size (cm)	1.85 × 0.75	3.49 × 1.22	4.79 × 1.67
Stem diameter (mm)	1.48 ± 0.26	2.70 ± 0.54	4.81 ± 0.83
Flower number	1.81 ± 0.74	6.00 ± 2.14	19.00 ± 8.41
Fruit‐bearing number	1.33 ± 0.75	3.88 ± 1.51	11.60 ± 3.93
Bearing rate	74.21 ± 34.77%	65.13 ± 16.53%	64.19 ± 8.83%
Fruit size (mm)	10.09 × 10.42	12.71 × 12.37	13.40 × 13.81
Seed number	1.27 ± 0.70	1.38 ± 0.63	1.70 ± 0.47
Monoembryony rate	92.00%	89.74%	90.00%

### Genomic and phylogenomic analysis of *F. hindsii*

To acquire a high‐quality *F. hindsii* genome sequence, we sequenced the most homozygous selfed line ‘S3y‐45’ by using PacBio single‐molecule technology. A total of ∼57.48 Gb PacBio data were produced, which were estimated to cover ∼145‐folds of *F. hindsii* genome. About 43.18 Gb of 10× genomic data and ∼51.25 Gb of Illumina data were generated to correct the PacBio assembly. By using Mecat, ARCS and LINKS, a ~374‐Mb genome assembly was finally obtained, which corresponds to ~96% of the predicted genome size and contains 1226 contigs and 900 scaffolds (contig‐N50 = 2.21 Mb, scaffold‐N50 = 5.16 Mb; Table 2). The remapping rate of the Illumina reads was 97.91% with a coverage rate of 98.50%. The error rate of assembly was lower than 0.01% as estimated by heterozygous SNP rate, indicating the accuracy of *F. hindsii* genome assembly. The completeness of the assembly was tested by 1369 eukaryotic genes in the Plantae BUSCO dataset to be 95.10%. The GC content of the genome was estimated to be 34.49%, and the GC content distribution (Figure S4) indicated the purity of the assembled genome. A total of 43.90% of the assembly sequence were identified as transposable elements. LTR/*Gyspy* was the most abundant repeat family, occupying 11.91% of the genome, followed by LTR/*Copia* (9.79%; Table S5). By *Ab initio* gene prediction, homology search and RNA‐seq analysis, 32 257 protein‐coding genes with 52 686 transcripts were identified in the genome assembly (Table 2). The average lengths of transcripts, coding sequences and exons were 2330 bp, 1268 bp and 371 bp, respectively. Collectively, the above results indicated the high quality and coverage of the *F. hindsii* genome assembly.

**Table 2:** Assembly and annotation statistics of *F. hindsii* genome

*F. hindsii* genome	
Size of assembled contig (bp)	373 557 569
Number of contigs	1226
Largest contig (bp)	11 998 049
Contig N50 (bp)	2 209 464
Contig N90 (bp)	75 418
Scaffold N50 (bp)	5 155 544
Scaffold N90 (bp)	124 411
GC content	34.49%
Number of gene models	32 257
Mean transcript length (bp)	2330
Mean coding sequence length (bp)	1268
Mean exon length (bp)	371
Percentage of transposable element	43.60%

To assess the nature of *F. hindsii* genome structure, we investigated the syntenic relationships of *F. hindsii*, sweet orange and pummelo genomes, for which a consecutive genome region containing at least five orthologous genes was defined as a syntenic block. For analysis of internal syntenic relationships, a total of 592 syntenic blocks containing 5128 genes were identified within *F. hindsii* genome. For intergenomic syntenic relationships, 1103 and 1141 syntenic blocks were, respectively, identified between *F. hindsii* and sweet orange and between *F. hindsii* and pummelo (Figure S5), which contained 19 693 and 13 690 orthologous genes. The above results indicated a high genomic collinearity between *F. hindsii* and other two citrus in genomes, confirming a conserved genome structure of citrus species (Wang *et al*., 2017; Xu *et al*., 2013). To further verify the gene conservativeness of *F. hindsii* among Citrinae species, we performed a comparative analysis between *F. hindsii* genome and other eight Citrinae genomes, including Chinese box orange (*Atlantia buxifolia*), pummelo (*Citrus grandis*), citron (*Citrus medica*), papeda (*Citrus ichangensis*), sweet orange (*Citrus sinensis*), satsuma mandarin (*Citrus unshiu*), wild mandarin (*Citrus reticulata ‘Mangshan’*) and clementine mandarin (*Citrus clementina*; Shimizu *et al*., 2017; Terol *et al*., 2016; Wang *et al*., 2018; Wu *et al*., 2014; Xu *et al*., 2013). In total, 230 523 genes were identified from the nine genomes and they were grouped into 27 493 gene families, of which 12 360 were shared by all the nine species. Among them, 262 gene families consisting of 986 genes were putatively specific to *F. hindsii* (Table S6) and 4003 *F. hindsii* orphan genes were not clustered into any family. The species‐specific genes were enriched (*P* ≤ 0.05 and FDR ≤ 0.05) in diverse biological processes, such as ‘organonitrogen compound metabolic process’, ‘small molecule metabolic process’ and ‘shoot apical meristem development’ (Table S7).

**Figure 2:**
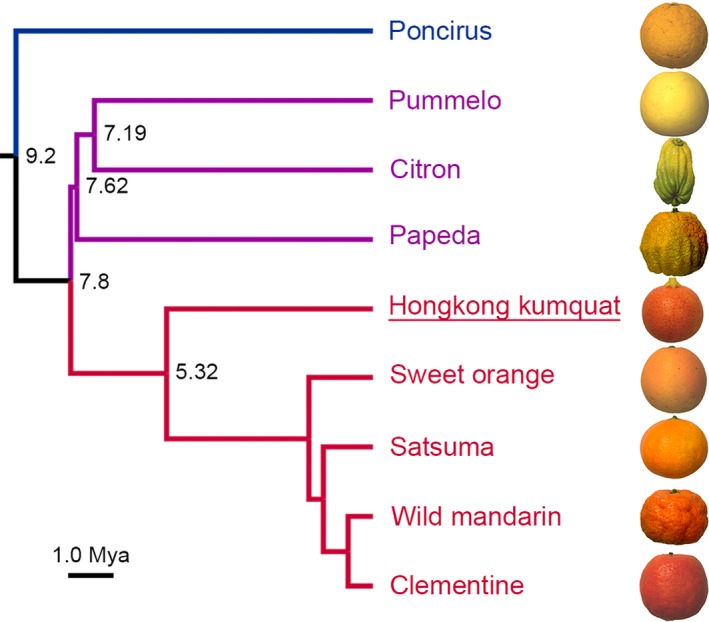
Phylogenetic Status of *F. hindsii* among Citrinae Group. The phylogenetic tree of nine Citrinae indicates their evolutionary relationships and estimated divergence time. Hongkong kumquat (*F. hindsii*) shows close relationship with mandarin species (*C. reticulata*). The tree topology was constructed by using 5848 conserved genes shared by these nine genomes. The number at each node denotes the estimated divergence time. Bar = 1.0 millions of years.

Although *F. hindsii* has been assigned to the *Fortunella* genus in Aurantioideae based on the morphology, pomological traits and molecular markers (Garcia‐Lor *et al*., 2013; Swingle, 1967; Tanaka, 1954), a more primitive phylogenetic status had been hypothesized due to its wide distribution in the wild and primitive fruit features (Yasuda *et al*., 2010). Thus, to further reveal the genetic relationship between *F. hindsii* and other citrus species, a phylogenetic tree was constructed based on the SNPs in 5848 conserved low‐copy genes in the above genomes (except for *Atlantia buxifolia*) and trifoliate orange genome (Data S1). The results showed three main clades (Figure 2): Clade I contained *P. trifoliata* (trifoliate orange) located at the basal position of the tree; Clade II included *F. hindsii*, all *C. reticulata* (mandarin) accessions and *C. sinensis* (sweet orange); and Clade III contained *C. grandis* (pummelo), *C. medica* (citron) and *C. ichangensis* (papeda). The tree topology and the estimated differentiation time indicated that *F. hindsii* is phylogenetically close to *C. reticulata*. The estimated divergence time suggested that the speciation of *F. hindsii* was not only later than the divergence of *Poncirus* and *Citrus* genus but also later than that of *C. grandis* and *C. reticulata*, which is in agreement with the results obtained by using SNPs, SSRs and indels (Garcia‐Lor *et al*., 2013; Wu *et al*., 2018). Taken together, the above results demonstrated the close phylogenic relationship between *F. hindsii* and *Citrus* genus, indicating the genomic and genetic potentials of *F. hindsii* in citrus research.

### Transcriptome profile of tissues in the seed‐to‐seed cycle

**Figure 3:**
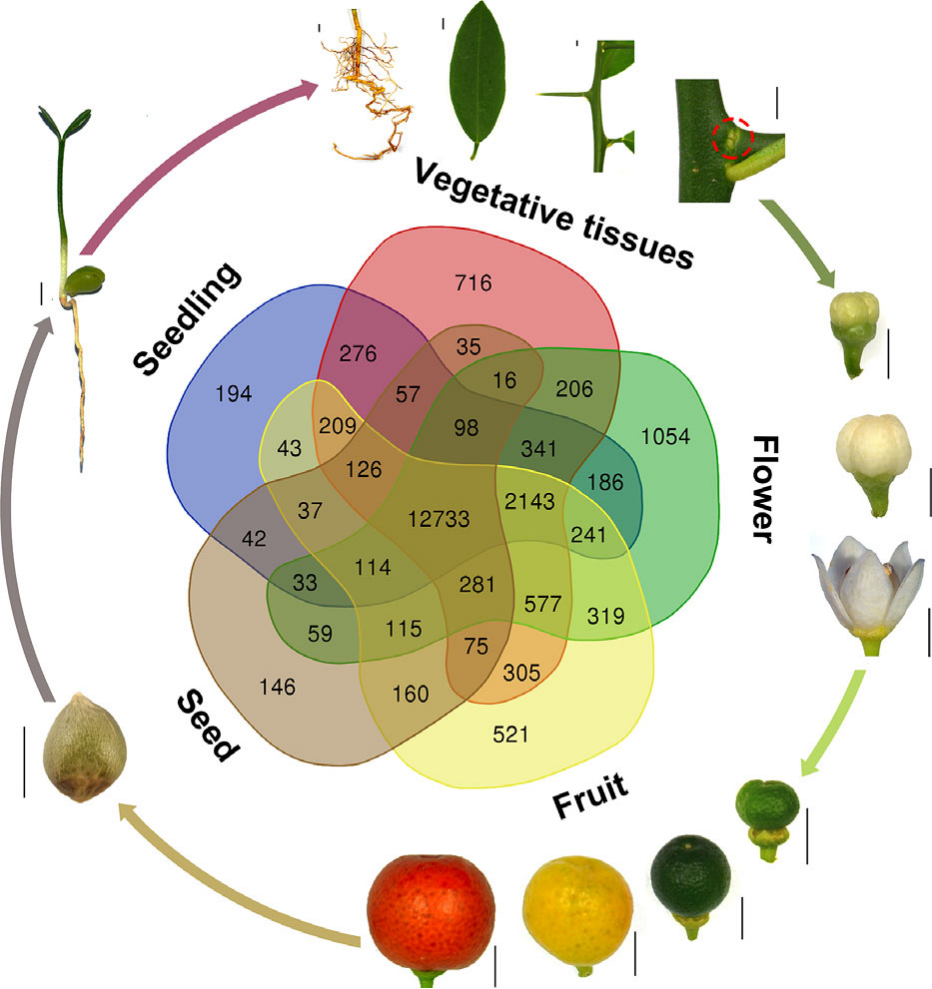
Life Cycle of *F. hindsii* and Gene Expression Patterns at Different Developmental Stages. The Venn diagram shows unique and shared gene numbers in the five organs (seed, seedling, adult vegetative, flower and fruit). The outer ring shows the 13 representative tissues used in WGCNA analysis. The red dashed cycle indicates the bud meristem tissue (Bar = 5 mm).

**Figure 4:**
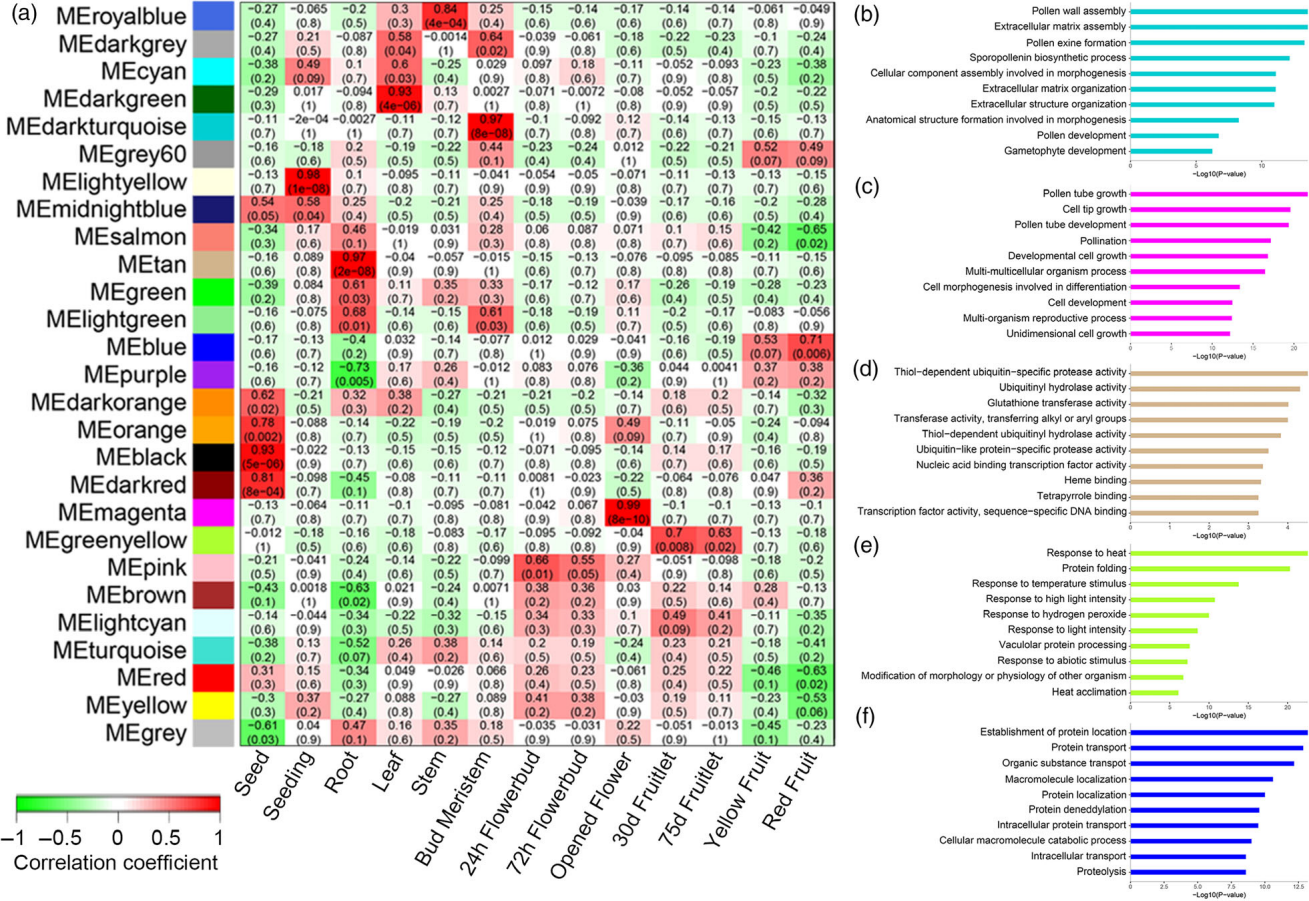
Detection of Co‐expression Network in Reprehensive Tissues of *F. hindsii*. (a) Module–tissue association matrix classifies the genes expressed in 13 tissues into 27 co‐expression modules. In the matrix, each row corresponds to a module and each column corresponds to a specific tissue. The colour of each cell at the row–column intersection indicates the correlation coefficient between the module and the tissue. A high degree of correlation between a specific module and the tissue type is indicated by dark red or dark green. (b) to (f) Gene ontology classifications of the genes in MEdarkturquoise, MEmagenta, MEtan, MEgreenyellow and MEblue modules, respectively.

To profile the gene expression pattern across *F. hindsii* life cycle, RNA sequencing was performed on 13 tissues from five organs (seed, juvenile seedling, adult vegetative tissues, flower and fruit; Figure 3). A total of 1548 billion Illumina reads were generated, 94.50% of which were mapped to the assembled genome. The expression levels of 26 780 genes (83% of total annotated genes) were detected and quantified in at least one tissue (Figure S6). In total, 12 798 genes were found to have common expression in all the five organs, suggesting their conserved functions in various tissues and developmental phases, and ‘Flower’ showed the largest number of specifically expressed genes (1007; Figure 3). In addition, weighted gene co‐expression network analysis (WGCNA) was carried out to identify the co‐expression patterns of genes across the 13 tissues. As shown in Figure 4a, the expression of all genes was clustered into 27 co‐expression modules, each of which was represented by its eigengene, namely the most notable component gene (Data S2). Obviously, seven modules showed high correlations with specific tissues (*r* > 0.8), such as MEdarkturquoise for bud meristem, MEmagenta for open flower and MEtan for root (Figure 4b–d), indicating that different biological processes dominate different organs or development phases. In addition, three modules showed medium correlations (*r* > 0.5) with certain tissues, such as MEgreenyellow for 30‐ and 75‐day fruit, and MEblue for yellow‐ and red‐mature fruit (Figure 4e–f), suggesting that they play certain roles in fruit enlargement and ripening.

**Figure 5:**
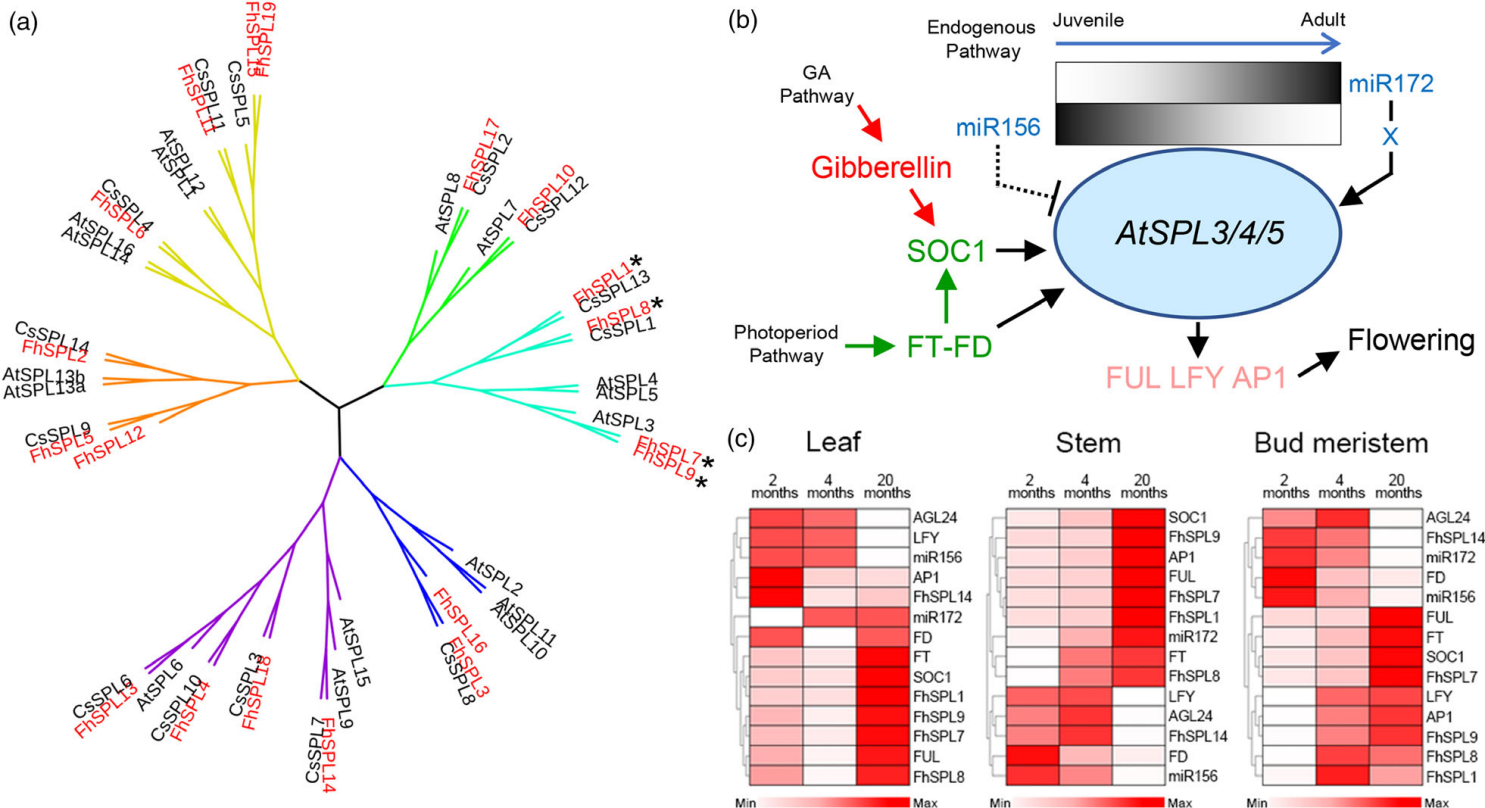
Phylogenetic Analysis of *FhSPLs* and Expression Patterns of *FhSPL1/7/8/9*. (a) Phylogenetic tree of *FhSPLs*,* CsSPLs* and *AtSPLs* based on the conserved SBP domain alignment of 19 predicted FhSPL proteins, 16 AtSPL proteins and 14 CsSPL proteins. The asterisks indicate the *AtSPL3/4/5* homologies of *F. hindsii*. (b) *AtSPL3/4/5* have been reported as a regulatory hub and signal amplifier as well as a key regulator in endogenous pathway involved in plant floral induction. (c) Expression patterns of *FhSPL1/7/8/9* (*AtSPL3/4/5* homologies) and their interacting flowering genes in leaf, stem and bud meristem tissues of 2 months (juvenile), 4 months (juvenile) and 20 months (adult) *F. hindsii* trees.

Among citrus species, *F. hindsii* showed very special floral characteristics, namely early and continuous flowering. As a very complicated and vital biological progress, flower development has been characterized as three consecutive and interrelated steps: floral induction, floral meristem identity and floral morphogenesis (Blazquez, 2000). To identify the candidate genes involved in the early flowering of *F. hindsii*, 86 genes involved in these three steps were selected (Table S8) based on their tissue‐specific expression, WGCNA results and the knowledge about flower development (Blazquez, 2000; Irish, 2010). Their expression levels in leaf and flower organs were compared between *F. hindsii* and pummelo, and between *F. hindsii* and lemon. Of these genes, 31 showed remarkably different expression (Fold Change >2 or <0.5) and 18 of them were involved in floral induction process (Figure S7 and Table S8), suggesting that a distinct floral induction pattern is possibly dominant in *F. hindsii*. Since *SQUAMOSA PROMOTER BINDING PROTEIN (SBP)‐LIKE (SPL)* transcription factors are involved in an endogenous flowering pathway (Fornara and Coupland, 2009; Jung *et al*., 2012; Wang *et al*., 2009), we performed a genome‐wide analysis of SPL genes in *F. hindsii*. In total, 19 genes with the SBP domain were identified in *F. hindsii* genome (Figure S8 and Table S9), which were more than those in *C. sinensis* (14) and *C. clementine* (15) (Liu *et al*., 2017b; Shalom *et al*., 2015). Phylogenetic analysis revealed that *AtSPLs*,* CsSPLs* and *FhSPLs* fell into six main clusters (Figure 5a), and four clusters (Cluster I, II, IV and V) included more *FhSPLs* than *CsSPLs*. The expression patterns of these *FhSPLs* showed both redundancy and specificity across 13 tissues (Figure S9), which was in agreement with the previous report about this gene family (Yamaguchi *et al*., 2009). Of these *FhSPLs*, the members in Cluster IV (*FhSPL*1/7/8/9) had homologous relationships with *AtSPL3/4/5*, which have been well characterized as both a key regulator in the endogenous flowering pathway and a signal amplifier or regulatory hub that joints photoperiod and gibberellin (GA) floral induction pathways (Figure 5b; Jorgensen and Preston, 2014; Jung *et al*., 2012; Wang *et al*., 2009; Wu *et al*., 2009; Xu *et al*., 2016; Yamaguchi *et al*., 2009). All the four *FhSPLs* in Cluster IV showed a high sequence homology with *AtSPL3/4/5*, could be translated to short‐type SPL proteins, and contained miR156 target sites in their 3′ UTR (Figure S8 and Table S10), suggesting that these *FhSPLs* have similar functions with *AtSPL3/4/5*. The qRT‐PCR experiment confirmed that *FhSPL1/7/8/9* had partially overlapped expression with each other, and their expression was correlated with that of the key flowering genes (Figure 5c), indicating the redundancy of their functions in floral induction. Meanwhile, in adult bud meristem, the expression of *FhSPL7* was significantly up‐regulated and positively correlated with that of FT, FUL and SOC1 but negatively correlated with that of miR156, suggesting that it plays a dominant role in floral induction among these four *SPLs*.

### Application of CRISPR/Cas9 technology on *F. hindsii*

**Figure 6:**
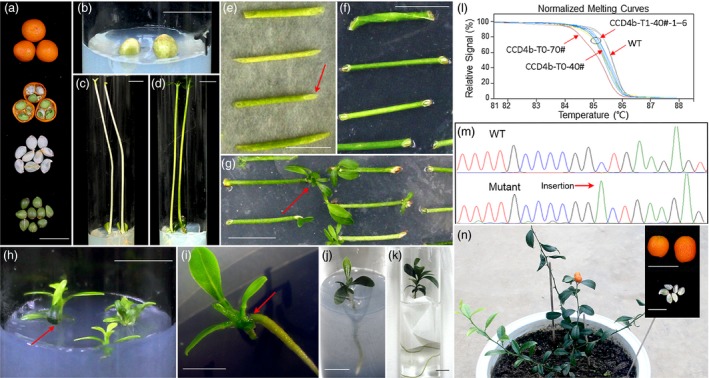
Flow chart of CRISPR‐based Targeted Mutagenesis in *F. hindsii* by Agrobacterium‐mediated Transformation. (a) The episperm of the collected seeds was removed, and the seeds were sterilized. (b) The sterilized seeds were cultured on MT medium. (c) The germinated seeds were cultured under darkness for 4 weeks. (d) The seedlings were cultured under a 16 : 8 photoperiod for regreening (10 days). (e) The epicotyl segments were co‐cultured with *Agrobacterium tumefaciens* under darkness (3 days). The red arrow indicates the cut surfaces. (f) The infected segments were cultured on shoot‐inducing medium under darkness (1 week). The red arrow indicates the cut surfaces that would produce white callus. (g) The shoots were induced from white callus under a 16 : 8 photoperiod (4 weeks). The red arrow indicates the regeneration of shoots from the white callus. (h) The regenerated shoots were cultured on root‐inducing medium under a 16 : 8 photoperiod. The red arrow indicates the cut surface and white callus. (i) The regenerated roots were induced from white callus (8 weeks). The red arrow indicates the regeneration of roots from the white callus. (j) to (k) Seedlings with vigorous roots were cultured in water for acclimatization (1 week) by using paper wick method. (l, m) The transgenic plants were genotyped via HRM analysis and Sanger sequencing. (n) Mature fruits and seed (T_1_) of T_0_ (about 9 months). Bars = 1 cm.

**Figure 7:**
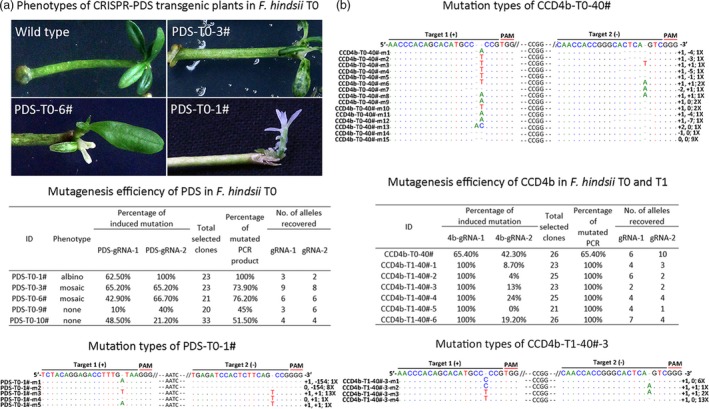
CRISPR/Cas9‐mediated Targeted Mutagenesis of *F. hindsii*. (a) Phenotypes, mutagenesis efficiency and mutation types of CRISPR‐mediated *FhPDS* transgenic plants in T_0_ generation. The alignment presents all the five mutation types of accession PDS‐T0‐1# (albino phenotype); the first and second number indicate the insertion (+) or deletion (−) of bases at target‐1 and target‐2, respectively; ‘X’ indicates the number of clones showing this genotype. (b) Characterization of CRISPR‐mediated CCD4b target mutagenesis in the first (T_0_) and subsequent (T1) generation of transgenic *F. hindsii*.

To further utilize *F. hindsii* for research purposes, we developed an efficient and practical CRISPR/Cas9 system based on *F. hindsii* via Agrobacterium‐mediated transformation (Figure 6). Firstly, to determine whether this system would work in *F. hindsii*, we designed two different sgRNAs specifically targeting the coding sequence of phytoene desaturase (PDS) gene, whose inactivation would result in the albino phenotype of leaves (Kaur *et al*., 2018; Odipio *et al*., 2017). In total, ten transgenic plantlets were regenerated from about 500 epicotyl segments (Figure S10), with five of them exhibiting targeted mutagenesis at the targeting sites in *FhPDS*. Among the five mutants, one showed global albino, two displayed mosaic albino and the other two showed no obvious abnormal phenotypes (Figure 7a). For the albino transgenic shoot (PDS‐T0‐1#), the mutation rate of the sgRNA1 targeting site was 62.5% with three kinds of mutant alleles and the sgRNA2 targeting site showed a mutation rate of 100% with two mutant alleles, generating an overall mutation rate of 100% for *FhPDS* (Figure 7a). For the two plantlets with mosaic albino, one showed mutation rates of 65.2% and 65.2% at sgRNA1 and sgRNA2 targeting sites, generating an overall mutation rate of 73.9% for *FhPDS*; the other showed mutation rates of 42.9% and 65.7%, generating a 76.2% overall mutation rate. For the two plantlets without albino, one showed mutation rates of 10.0% and 40.0% at the two targeting sites, inducing an overall mutation rate of 45.0%; the other showed mutation rates of 48.5% and 21.2%; and the overall mutation rate was 51.5% (Figure 7a). The major mutation type in all the above CRISPR‐modified transgenic *F. hindsii* was also 1‐bp insertion (Table S11), which was consistent with CRISPR‐induced target mutagenesis in rice and *Arabidopsis* (Ma *et al*., 2015b). We also assessed five potential off‐target sites in this system using Sanger sequencing of PCR amplicons, and the results showed that no mutation was detected in these potential off‐target sites (Table S12).

Furthermore, since the dysfunction of *PDS* gene would severely affect the vitality of plants, we constructed another CRISPR/Cas9 vector targeting CAROTENOID CLEAVAGE DIOXYGENASE4 (CCD4) to validate whether this system would be stably inherited by subsequent generations in *F. hindsii*. Two positive T_0_ seedlings (CCD4‐T0‐40# and CCD4‐T0‐70#) carrying the target mutagenesis were obtained by the same method as CRISPR‐induced PDS mutant. Like the PDS mutants, the dominant mutation type of the T_0_‐CCD4b mutants was also 1‐bp insertion (Figure 7b). Fifteen alleles were observed in the T_0_‐CCD4b accession CCD4b‐T0‐40# (Figure 7b). However, because of the veiled function of CCD4b and no visible phenotype of CCD4b‐T0‐40#, it cannot be sure the wild‐type allele of T_0_ (CCD4b‐T0‐40#‐m15) was either derived from chimeric mutation or escaped cells (Figure 7b). Intriguingly, all six T_1_ lines (CCD4b‐T1‐40#‐1 to CCD4b‐T1‐40#‐6) from the T_0_ line CCD4b‐T0‐40# showed 100% overall mutation rate and harboured no wild‐type allele (Figure 7b). One of them (CCD4b‐T1‐40#‐3) showed a mutation rate of 100% at the sgRNA1 targeting site with two mutant alleles, and the other five T_1_ lines showed a mutation rate of 100% at the sgRNA1 targeting site with at least three mutant alleles. Most mutation types in the CCD4b target sites of the T_1_ generations were the same to those of their parent T_0_, and there was also a new insertion of single nucleotide ‘C’ in the T_1_ generation (Figure 7b). Besides, we further developed a simplified high‐resolution melting (HRM) strategy to perform target genotyping, which could clearly identify the CRISPR/Cas9‐induced micro‐indels and SNPs. As shown in Figure 6, the HRM results of CCD4b‐T0‐40# and CCD4b‐T0‐70# transgenic plants were consistent with their Sanger‐sequencing results (Figure 6l, m). Besides, the HRM curves of CCD4b‐T1‐40#‐1–6 were better fitted with that of CCD4b‐T0‐40# than with those of CCD4b‐T0‐70#, suggesting a high distinguishability of this HRM method.

In summary, these results demonstrated that the CRISPR/Cas9 system can be efficiently applied on *F. hindsii* and a more stable T_1_ could be induced based on this system. More importantly, it only took about 5 months to generate the T_0_ plantlets from the seeds and about another 10 months to obtain subsequent T_1_ generation with a target mutation rate of 100% (Figure 6n), demonstrating the promising efficiency of this system in saving time and resources in the studies of citrus gene functions.

## Discussion

For citrus genetic research, the previously used monoembryonic species/varieties usually have long periods of juvenility, such as 6–10 years for pummelo and citron (Aleza *et al*., 2010b). Besides, most modern citrus species, such as sweet orange, mandarin and lemon, have the inherent characteristic of polyembryony. Thus, owing to its advantages of monoembryony, short juvenility and small canopy, *F. hindsii* shows unique superiority as an excellent time‐ and resource‐saving material for citrus genetic mapping. For example, a genetic population with 220 hybrids between *F. hindsii* and *F. crassfolia* has been successfully bred and cultivated on a just 10 m^2^ horticultural shelf. Based on this population, a fine genetic map for fruit‐related traits has been under construction in our laboratory. In addition, a juvenile period of 8 months of *F. hindsii* is the shortest among the early flowering germplasms in the citrus taxa to our knowledge (Tong *et al*., 2009), suggesting the prospects of generating BC1, F2 or even introgression lines based on *F. hindsii*. More importantly, all previous transgenic *F. hindsii* lines carrying different sequences blossomed and bore fruits in their first or second year (Cao *et al*., 2015; Liu *et al*., 2017c; Zhang *et al*., 2009), suggesting that genetic modification does not disturb its intrinsic feature of short juvenility. Thus, *F. hindsii* is promising in seed‐to‐seed propagation within 1 year for diverse research purposes just like in annual model plants. However, the efficiency of Agrobacterium‐mediated method on *F. hindsii* is not very high so far (0.2%–4%, from explant to positive transgenic plantlet, empirically). In addition, although our sequenced accession S3y‐45 was relatively homozygous, a certain extent of inbreeding depression was observed on it, such as abnormal stamens and sepals. Thus, improvement of the transformation method and selection of optimal materials from the S_1_ and S_2_ populations are being carried out in our laboratory.

In this study, we presented the high‐quality genome of *F. hindsii*, which is the first genome sequence of *Fortunella* genus and the most consecutive Citrinae genome to date (contig‐N50 = 2.21 Mb, completeness = 98.50%). Characterization of the *F. hindsii* genome for protein‐coding genes and comparative genomic analysis revealed that about 96.9% of *F. hindsii* genes have homologues in Citrinae species, indicating the great potentials of this germplasm to be used for gene function studies. Besides, as indicated by WGCNA results, the first spatio‐temporal gene co‐expression analysis covering the whole life cycle of *F. hindsii* depicted a transcriptomic profile of its growth and development, which is worth of further exploration for various research purposes. For example, in the present study, we were particularly interested in the mechanism underlying the unique inflorescence characteristics of *F. hindsii*. By a comparative transcriptomic analysis, we found that the key genes involved in photoperiod pathway, such as *FT*,* SOC1* and *AGL6*, were down‐regulated compared with in pummelo and lemon, whereas three flowering suppressor genes in vernalization pathway, *FLC*,* FLD* and *FWA*, whose inactivation relies on low‐temperature signal, were significantly up‐regulated. Thus, we turned to focus on the SPL transcription factors involved in an endogenous floral induction pathway. Four candidate genes were selected for further functional verification, providing a scenario of *F. hindsii* inflorescence regulation, which is less responsive to exogenous signals but dominated by SPL genes. This scenario is exactly in accordance with the ecological niche (low latitude and altitude, and under tree crown) and the inflorescence characteristics (later anthesis, continuous inflorescence and short juvenility) of *F. hindsii*. However, since SPL genes always have redundant functions in a same biological process and are well known for neo/sub‐functionalization in evolution (Preston and Hileman, 2013), it can be anticipated that gene editing technique would be an irreplaceable tool kit closely following data mining for revealing the exact roles of these four candidates and elucidating the functional differentiation between *CsSPL1/13* and *FhSPL1/7/8/9* in the future.

Despite that CRISPR/Cas9‐mediated target mutagenesis has been reported in sweet orange (Zhang *et al*., 2017) and grapefruit (Jia *et al*., 2017), no T_1_ generation of citrus species had been obtained due to the polyembryony and long juvenility. Here, we for the first time reported a practicable CRISPR/Cas9 system based on *F. hindsii*, which only needs about 15 months to generate T_1_ generation. However, the editing efficiency (about 50%) was not high when compared with that in previous studies (Jia *et al*., 2017; Peng *et al*., 2017; Zhang *et al*., 2017), which may be attributed to the vector elements and/or the nature of *F. hindsii* itself. Thus, along with the progress in the research on various gene functions, optimization of the CRISPR vectors has been under testing in our laboratory, such as the utilization of more appropriate U3/U6 promoters. More important, all T1 seedlings showed a mutation rate of 100% at the sgRNA1 targeting site, namely no wild‐type allele was detected in T1. This result indicates the reproduction process would overcome the interference from escaped cells in citrus T0, suggesting a more appropriate application of this *F. hindsii*‐based CRISPR/Cas9 system for citrus gene function study in future. HRM analysis has been widely applied in SNP detection and mutagenesis genotyping in animals and humans (D'Agostino *et al*., 2016; Dobrowolski *et al*., 2009; Dufresne *et al*., 2006), but was seldom applied on plants (Denbow *et al*., 2017). Here, we proposed an effective and economical HRM genotyping method for the identification of citrus mutagenesis, which demonstrates great potentials of further application in plant research. Taken together, based on *F. hindsii* which is characterized by short juvenility and monoembryony, we presented a novel solution to better apply CRISPR in citrus research, which may significantly shorten the time for the acquisition of T_0_ and T_1_ mutants.

In conclusion, *F. hindsii*, along with its high‐quality genome sequence and feasible CRISPR system, provides a potential ‘model citrus’ for citrus genomic and genetic studies.

## Methods

### Plant materials, selfing project and cultivation experiment

The scions of nine monoembryonic *F. hindsii* (S_0_ generation) were collected from the wild in our first survey at Longyan county, and their axillary buds were grafted to trifoliate orange outdoor at Huazhong Agricultural University in 2011 (N30.5, E114.4). For selfing, each plant was covered by a special screen cage during their anthesis. The harvested seeds were sown in plastic pot containing a potting mix of a commercial medium and perlite (ratio 3 : 1, v/v). One‐year‐old trees were cultivated in a heliogreenhouse with temperature ranging from 10 to 35 °C without any special treatment. The SSR markers used for preliminary estimation of homozygosity were shown in Table S13. For morphologic and phenological observations, seeds of accession SY01 were used; the morphological data were recorded during 2014–2017. The genome DNA for sequencing was extracted from the leaves of accession S3y‐45 by special CTAB methods (Cheng *et al*., 2003). The 13 representative tissues were sampled from seed, juvenile seedling, leaf, stem, root, bud meristem, 24‐h flower bud, 72‐h flower bud, open flower, 30‐day fruitlet, 75‐day fruitlet, yellow‐mature fruit (about 120 days) and red‐mature fruit (about 150 days). The tissues for RNA‐seq were sampled from accession SY02‐02 (seed and seedling; grand parent of S3y45) and S2y‐26 (the remaining tissues; parent of S3y45), with two biological replicates for each tissue (details see Figure S6). The transgenic T_0_ seedlings were cultivated in sterile room under 16 : 8 (day : night) photoperiod and at 26 °C air temperature.

### Genome assembly, annotation and phylogenic analysis

Single‐molecule long reads were generated by PacBio Sequel platform. Microfluidic partitioning of genomic DNA (gDNA) was performed using 10× Genomics Chromium System. Mecat was used for *de novo* assembly (Xiao *et al*., 2017), and the detailed assembly workflow was shown in Figure S11. For TE annotation, a *de novo* repeat library was constructed and annotated by RepeatModeler (Smit and Hubley, 2008). Then, the library was integrated based on RepBase (Bao *et al*., 2015) database. RepeatMasker was finally used to identify the repeat elements in the genome.

Gene models were predicted by combining *ab initio* gene prediction, homology sequence analysis and the RNA‐seq results. Augustus (Stanke *et al*., 2006) and GlimmerHMM (Majoros *et al*., 2004) were employed to perform *ab initio* prediction for gene models. Then, the gene structure was further confirmed based on published protein and expressed sequence tags (EST) of citrus species by using AAT (Haas *et al*., 2008) and Exonerate (Slater and Birney, 2005). All RNA‐seq reads were aligned to the assembled genome using Tophat2 (Kim *et al*., 2013), and the alignments were imported into Cufflinks (Trapnell *et al*., 2012) for transcript assembly. The genome‐guided and *de novo* transcript assembly was performed by Trinity (Grabherr *et al*., 2011). RNA‐seq assemblies were further refined by using PASA (Haas *et al*., 2003). All the predicted gene structures were integrated by EVM (Haas *et al*., 2008). The gene models were then updated by PASA assembly alignments. The annotated proteins were aligned to the SwissProt and TrEMBL databases (Consortium, U.P, 2015) by using Blastp (*E*‐value < 10^e−6^). The functional information of the best matched protein was extracted. The motifs and domains within the gene models were identified by InterProScan (Mulder and Apweiler, 2007) against multiple public databases (ProDom, PROSITE, PRINTS, Pfam, PANTHER and SMART). The gene ontology IDs were obtained from the corresponding InterPro entry (Mulder and Apweiler, 2007).

### Transcriptome sequencing, data analysis and qRT‐PCR experiment

Total RNA of all the tissues was extracted by using TRIzol reagent (Takara). Raw RNA‐seq data were processed to remove low‐quality reads by using Trimmomatic (Bolger *et al*., 2014). The expression level was evaluated by normalization to FPKM value calculated from the number of aligned reads for each gene. Differential expression analyses were performed by using Deseq2 (Love *et al*., 2014). Genes with adjusted *P*‐values lower than 0.05 and at least twofold expression changes were defined as differentially expressed genes (DEGs). The correlation between replicates was analysed by using R package. GO enrichment analysis was performed by the web‐based agriGO (Tian *et al*., 2017) with the annotation data of assembled genome as the statistical background. WGCNA package (Zhang and Horvath, 2005) was used for co‐expression analysis, for which genes expressed (FPKM ≥ 2) at least in one tissue were used as the input data. The expression data of pummelo and lemon were obtained from a previous project (Terol *et al*., 2016). The citrus orthologs of the selected flowering genes were revised based on local NCBI‐Blast and the genome annotation results. The amino acid sequence and gene structure data of *AtSPLs* were downloaded from PlantTFDB (Jin *et al*., 2017; http://planttfdb.cbi.pku.edu.cn/). HMMER 3.0 (Prakash *et al*., 2017) was used to search the SBP domain from the *F. hindsii* and sweet orange genomes (http://citrus.hzau.edu.cn/orange/). The predicted *FhSPLs* were further confirmed by PFAM (Finn *et al*., 2016) and SMART (Letunic and Bork, 2018). The phylogenetic tree of the SPL genes was constructed by MEGA 7.0 (Kumar *et al*., 2016) based on the amino acid sequences of the highly conserved 76‐bp SBP domain (Data S3). The prediction of motifs, conserved domains and miRNA‐targets was performed by online tools MEME (http://meme-suite.org/), NCBI‐CCD (https://www.ncbi.nlm.nih.gov/cdd) and psRNATarget (http://plantgrn.noble.org/psRNATarget/), respectively.

The qRT‐PCR experiment was carried out on the three successive development phases (2, 4 and 20 months) of leaf, stem and bud meristem, with three biological replicates for each tissue. The cDNA libraries were constructed by using HiScript II QRT SuperMix for qPCR (Vazyme, R223‐01), and miRNA‐cDNA libraries were constructed by using the stem‐loop reverse transcription method (Varkonyi‐Gasic *et al*., 2007). The RT‐PCR reaction was performed on *Roche* (Indianapolis, IN) LightCycler^®^ 480, following the manufacturer's instructions of LightCycler^®^ 480 SYBR Green I Master mix (Vazyme). The reactions were carried out with the cycling profile of 95 °C for 30 s, followed by 45 cycles of 95 °C/10 s, 60 °C/30 s and 72 °C/10 s. *Roche* LightCycler 480 software version 1.5.1.62 was used to perform data analysis, and the relative gene expression values were calculated by using the 2^−▵▵CT^ method. ACTIN and U6 were used as the internal reference for quantifying the flowering genes and miRNAs, respectively. All used primers in this experiment were shown in Table S14.

### CRISPR/Cas9 vector construction, genotyping of mutagenesis and HRM analysis

The binary vector contained two copies of CaMV 35S promoter, which would drive Cas9 (optimized with plant codon) and phosphotransferase II (NPTII) gene expression, respectively (Ma *et al*., 2015b). The sgRNAs were designed by CRISPR‐P web tool (Liu *et al*., 2017a). The overlapping PCR and Gibson assembly method (Gibson *et al*., 2009) were used to construct a gRNA cassette which would express two guiding RNA sequences for targeting the purposed gene (details see Figure S12). The two gRNAs targeting *FhPDS* were spaced with 400 bp, and the other two targeting *FhCCD4b* were spaced with 100‐bp interval. We used CRISPR web tool (http://crispr.hzau.edu.cn/CRISPR/) to predict the off‐target sites (off‐score > 0.09 as criterion), and the summary of putative off‐target analysis is shown in Table S12. The primers used for detection of off‐target, vector construction, sequencing and HRM genotyping were all displayed in Table S15. The PCR reaction of HRM was performed on *Roche* (Indianapolis, IN) LightCycler^®^ 480 following the manufacturer's instructions of LightCycler^®^ 480 High Resolution Melting Master mix. RT‐PCR reactions were carried out following the cycling profile of 95 °C for 2 min, followed by 45 cycles of 95 °C for 10 s, 65 °C for 10 s and 72 °C for 10 s. The genotyping of HRM was carried out following 95 °C for 1 min, 40 °C for 1 min, 65 °C for 1 min, 95 °C for 10 s and cooling to 40 °C. The fluorescence data of HRM were continuously acquired by 25 acquisitions per centigrade degree.

## Availability

Genome and transcriptome sequencing data have been deposited in NCBI database under BioProject PRJNA487160 and PRJNA497956, respectively. The assembled genome has been deposited in DDBJ/ENA/GenBank under the accession number QWBT00000000 and at http://citrus.hzau.edu.cn/orange/.

## Funding

The research was financially supported by the National Key Research and Development Program of China (Nos. 2018YFD1000106) and the National Natural Science Foundation of China (31630065 and 31521092).

## Conflict of interest

The authors declare no conflict of interest.

## Supporting information


**Figure S1** Outstanding Selfed *F. hindsii* and Their K‐mer Distributions.
**Figure S2** The Sequenced Accession ‘S3y‐45’, the Most Homozygous Selfed *F. hindsii*.
**Figure S3** The Tree Characteristics of *F. hindsii*.
**Figure S4** GC Content Distribution of *F. hindsii* Genome Assembly.
**Figure S5** Syntenic Relationships of *F. hindsii*, Sweet orange and Pummelo Genomes.
**Figure S6** Hierarchical Clustering of the Expression of the 13 *F. hindsii* Tissues.
**Figure S7** Expression Pattern of Genes Involved in Flowering in *F. hindsii*, Pummelo and Lemon.
**Figure S8** Genome‐wide SPL Identification of *F. hindsii*.
**Figure S9** Expression Pattern of *FhSPLs*.
**Figure S10** Positive Identification of Transgenic *F. hindsii* by PCR amplification.
**Figure S11** Brief Workflow of Genome Sequencing and Assembly in Present Study.
**Figure S12** Diagram of CRISPR/gRNA Vector Construction via Overlap‐PCR and Gibson Assembly.
**Table S1** Summary of *F. hindsii* Germplasm Collection.
**Table S2** Summary of *F. hindsii* Selfing Lines.
**Table S3** Summary of Genome Survey of Outstanding *F. hindsii* Selfed Offspring.
**Table S4** Estimation of *F. hindsii* Genome Size via Flow Cytometry.
**Table S5** TE Classification of *F*. *hindsii* Genome.
**Table S6** IDs of *F*. *hindsii* Specific Genes.
**Table S7** GO Enrichment Analysis of Genes Specific to *F. hindsii*.
**Table S8** The Flowering Gene Selected for Expression Analysis.
**Table S9** Gene IDs of 19 Identified *FhSPLs*.
**Table S10** Predicted miRNA Target‐side of Candidate *FhSPLs*.
**Table S11** Rate of CRISPR/gRNA Modified Various Nucleotide Insertion (+) and Deletion (‐) Events in *F. hindsii* Transgenic Lines.
**Table S12** Summary of Putative Off‐target Analysis of CRISPR/Cas9‐transgenic *F. hindsii*.
**Table S13** The SSR Markers Used for Homozygosity Estimation.
**Table S14** Primer Used for qRT‐PCR Experiment.
**Table S15** Primers Used in CRISPR Experiment.

**Data S1** Conserved Low‐copy Genes Used for Phylogenomic analysis.

**Data S2** Notable Component Genes of the 27 Co‐expression Modules.

**Data S3** Alignment of SBP Domain Used in Phylogenetic Analysis of SPL Genes.
