# Supplementary material for: Genome sequencing and CRISPR/Cas9 gene editing of an early flowering Mini‐Citrus (Fortunella hindsii)
Source: Plant Biotechnol J. 2019 May 21;17(11):2199–210. doi: 10.1111/pbi.13132 (PMC6790359; doi:10.1111/pbi.13132)
Supplement: Supplementary file 1 — Figure S1 Outstanding Selfed F. hindsii and Their K‐mer Distributions. Figure S2 The Sequenced Accession ‘S3y‐45’, the Most Homozygous Selfed F. hindsii. Figure S3 The Tree Characteristics of F. hindsii. Figure S4 GC Content Distribution of F. hindsii Genome Assembly. Figure S5 Syntenic Relationships of F. hindsii, Sweet orange and Pummelo Genomes. Figure S6 Hierarchical Clustering of the Expression of the 13 F. hindsii Tissues. Figure S7 Expression Pattern of Genes Involved in Flowering in F. hindsii, Pummelo and Lemon. Figure S8 Genome‐wide SPL Identification of F. hindsii. Figure S9 Expression Pattern of FhSPLs. Figure S10 Positive Identification of Transgenic F. hindsii by PCR amplification. Figure S11 Brief Workflow of Genome Sequencing and Assembly in Present Study. Figure S12 Diagram of CRISPR/gRNA Vector Construction via Overlap‐PCR and Gibson Assembly. Table S1 Summary of F. hindsii Germplasm Collection. Table S2 Summary of F. hindsii Selfing Lines. Table S3 Summary of Genome Survey of Outstanding F. hindsii Selfed Offspring. Table S4 Estimation of F. hindsii Genome Size via Flow Cytometry. Table S5 TE Classification of F. hindsii Genome. Table S6 IDs of F. hindsii Specific Genes. Table S7 GO Enrichment Analysis of Genes Specific to F. hindsii. Table S8 The Flowering Gene Selected for Expression Analysis. Table S9 Gene IDs of 19 Identified FhSPLs. Table S10 Predicted miRNA Target‐side of Candidate FhSPLs. Table S11 Rate of CRISPR/gRNA Modified Various Nucleotide Insertion (+) and Deletion (‐) Events in F. hindsii Transgenic Lines. Table S12 Summary of Putative Off‐target Analysis of CRISPR/Cas9‐transgenic F. hindsii. Table S13 The SSR Markers Used for Homozygosity Estimation. Table S14 Primer Used for qRT‐PCR Experiment. Table S15 Primers Used in CRISPR Experiment. [file PBI-17-2199-s003.docx]

**Supplemental Figures and Tables**

**
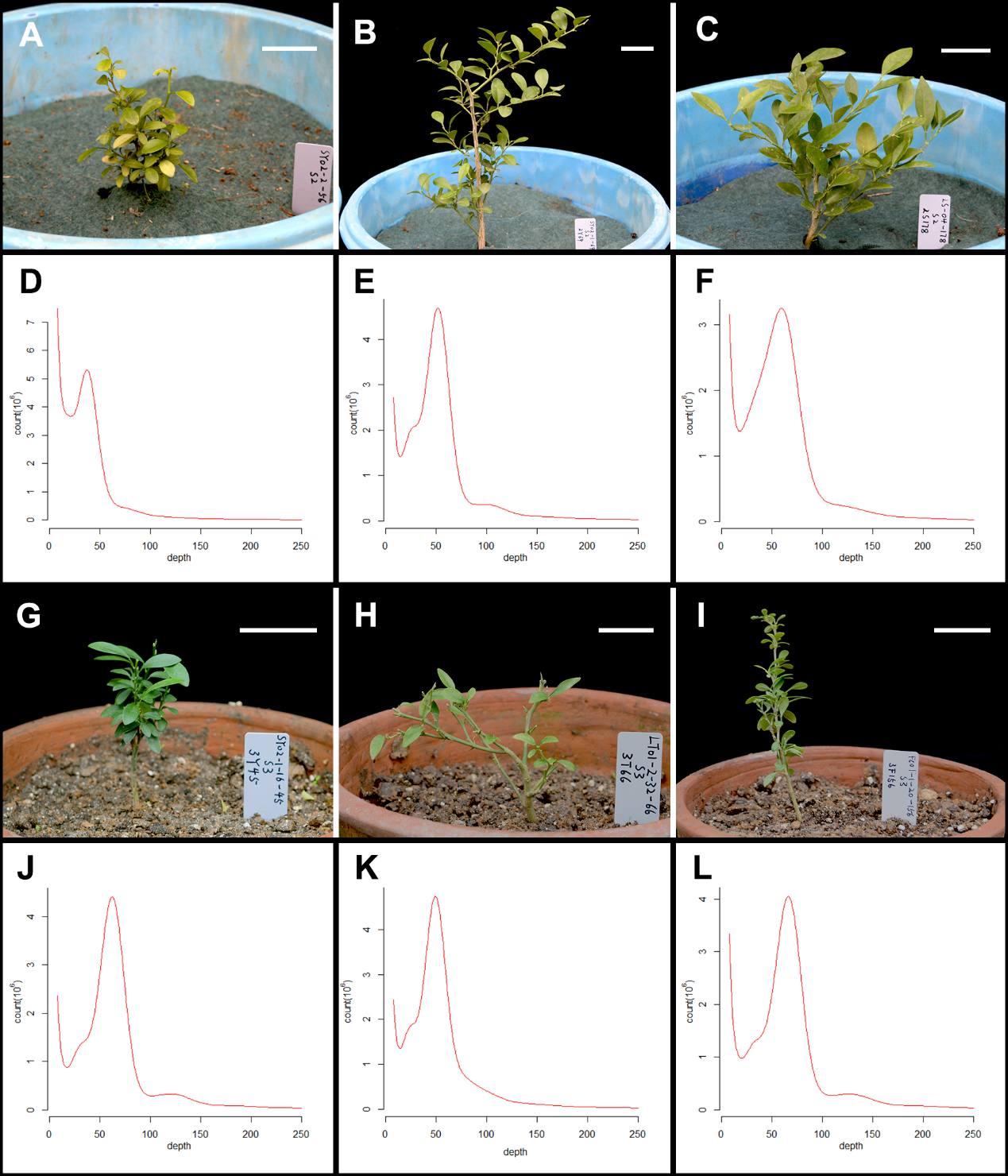
**

**Figure S1. Outstanding Selfed *F. hindsii* and Their K-mer Distributions.**

**(A)** and **(D)**, S2y-56 and its k-mer distribution.

**(B)** and **(E)**, S2y-69.

**(C)** and **(F)**, S2s-178.

**(G)** and **(J)**, S3y-45.

**(H)** and **(K)**, S3t-66.

**(I)** and **(L)**, S3t-66.

The frequency of each 17-mer was calculated based on the filtered paired-end reads. Bars = 2cm.

**
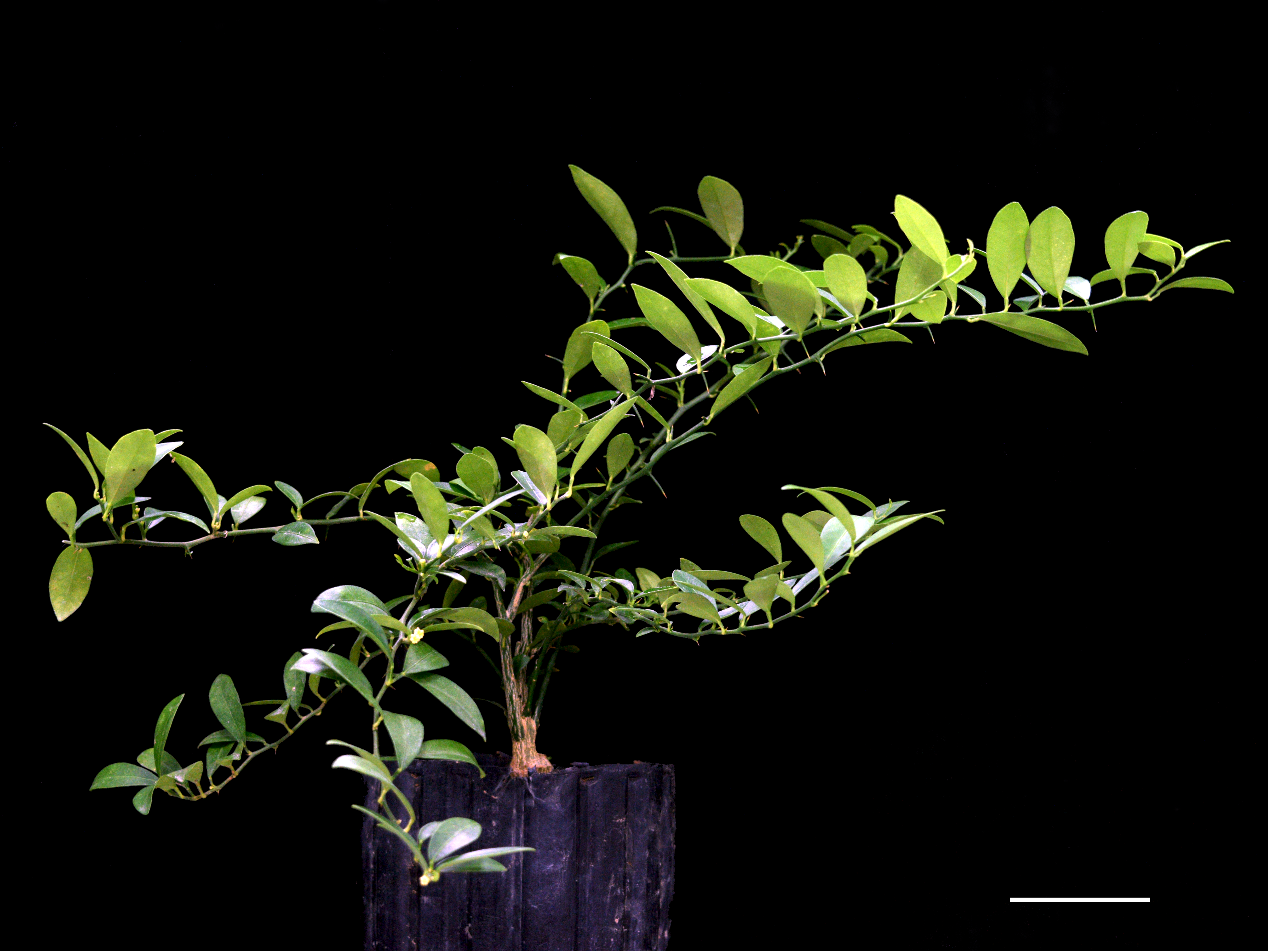
**

**Figure S2. The** **Sequenced Accession “S3y-45”, the Most Homozygous selfed *F. hindsii*.**

This is a grafted clone of S3y-45. The root stock is [trifoliate](javascript:;) [orange](javascript:;). The tree is two years old. Bar = 5cm.


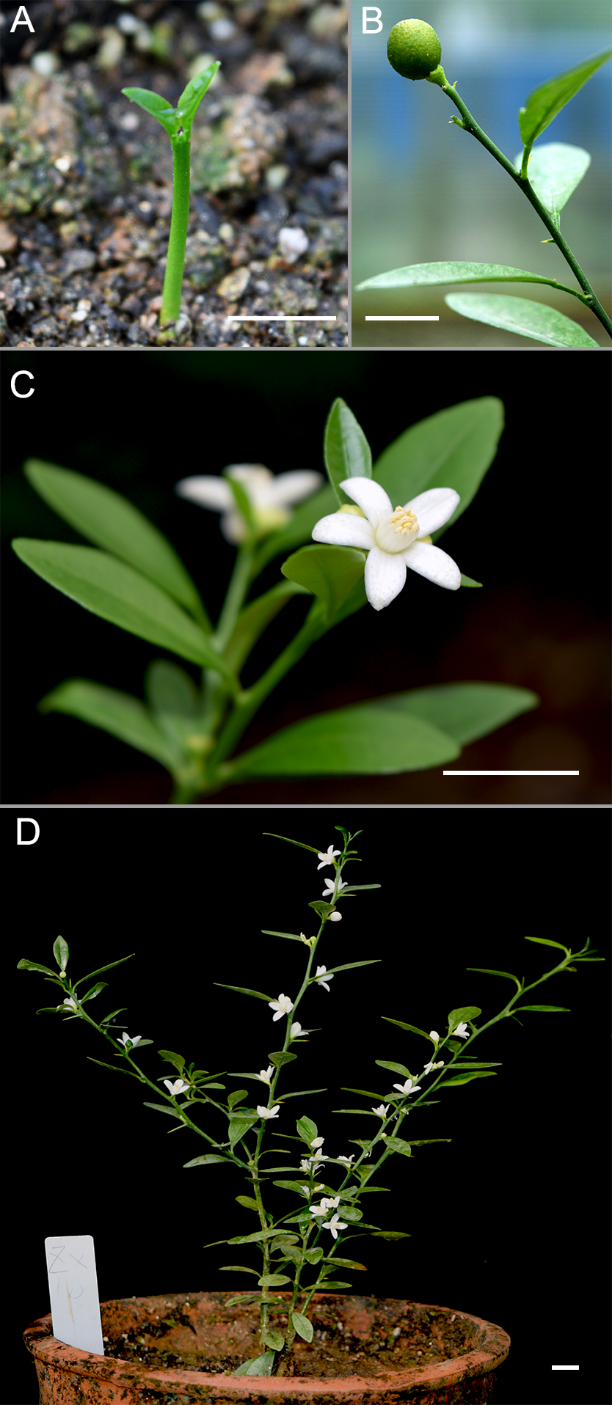


**Figure S3.** **The Tree Characteristics of *F. hindsii*.**

**(A)** Germination of a *F. hindsii* seeding.

**(B)** Fruit bearing of a one-year-old tree.

**(C)** Flowering morphology of a two-years-old tree; noting that the flowers developed on tender twigs.

**(D)** Flowering of a three-years-old *F. hindsii*.

**
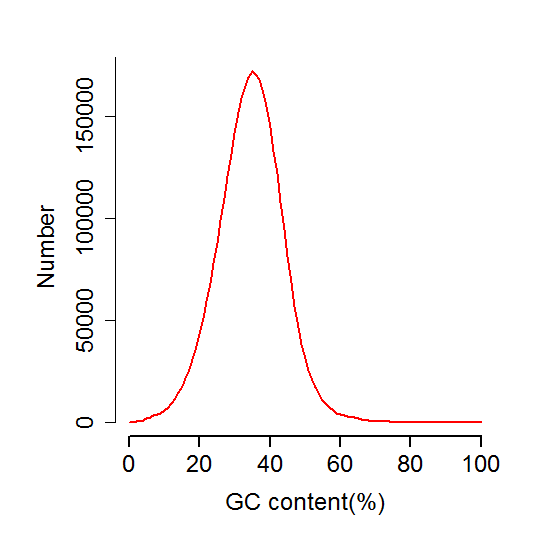
**

**Figure S4. GC Content Distribution of *F. hindsii* Genome Assembly.**

The peak of this curve is at 34.49%.

**
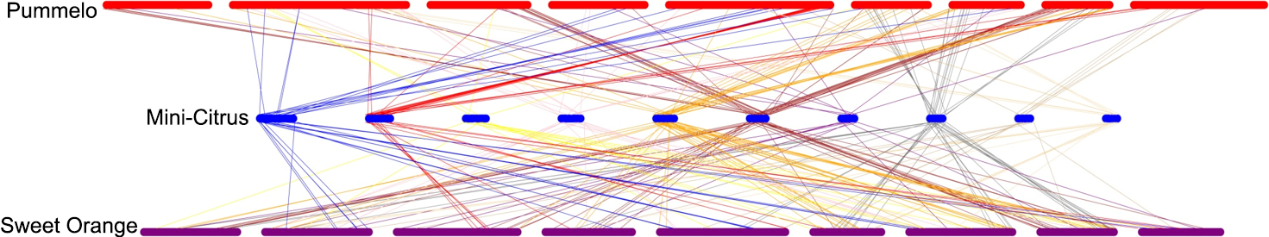
**

**Figure S5. Syntenic Relationships of *F. hindsii*, Sweet orange and Pummelo Genomes.**

Red and purple bars represent the nine chromosomes of pummelo and sweet orange, respectively. Blue bars represent the ten longest contigs of *F. hindsii* genome assembly. Each fine line indicates a linked relationship of two syntenic regions.

**
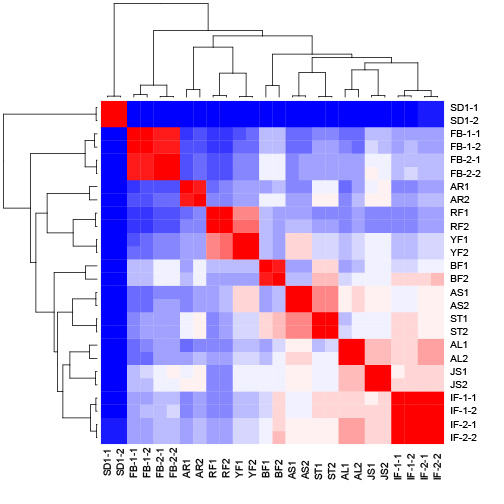
**

**Figure S6. Hierarchical Clustering of the Expression Data of the 13 Tissues from *F. hindsii*.**

Heat map shows the correlation among the 13 tissues and the repeatability between biological replicates. A high degree of correlation is indicated by dark red color.

SD is seed; FB-1 is 24-hours flower-bud; FB-2 is 72-hours flower-bud; AR is root; RF is red-mature fruit; YF is yellow-mature fruit; BF is opened flower; AS is stem; ST is bud meristem; AL is adult leaf; JS is seedling; IF-1 is 30-days immature fruit; IF-2 is 75-days immature fruit.

The FB-1, FB-2, RF, YF, BF, AS, ST, AL, IF-1 and IF-2 tissues were sampled from two biological replicates of S2y-26 (two grafted clones). The SD and JS tissues were sampled from two biological replicates of SY02-02 (two grafted clones). The RT1 and RT2 tissues were sampled from S2y-26 and SY02-02, respectively.


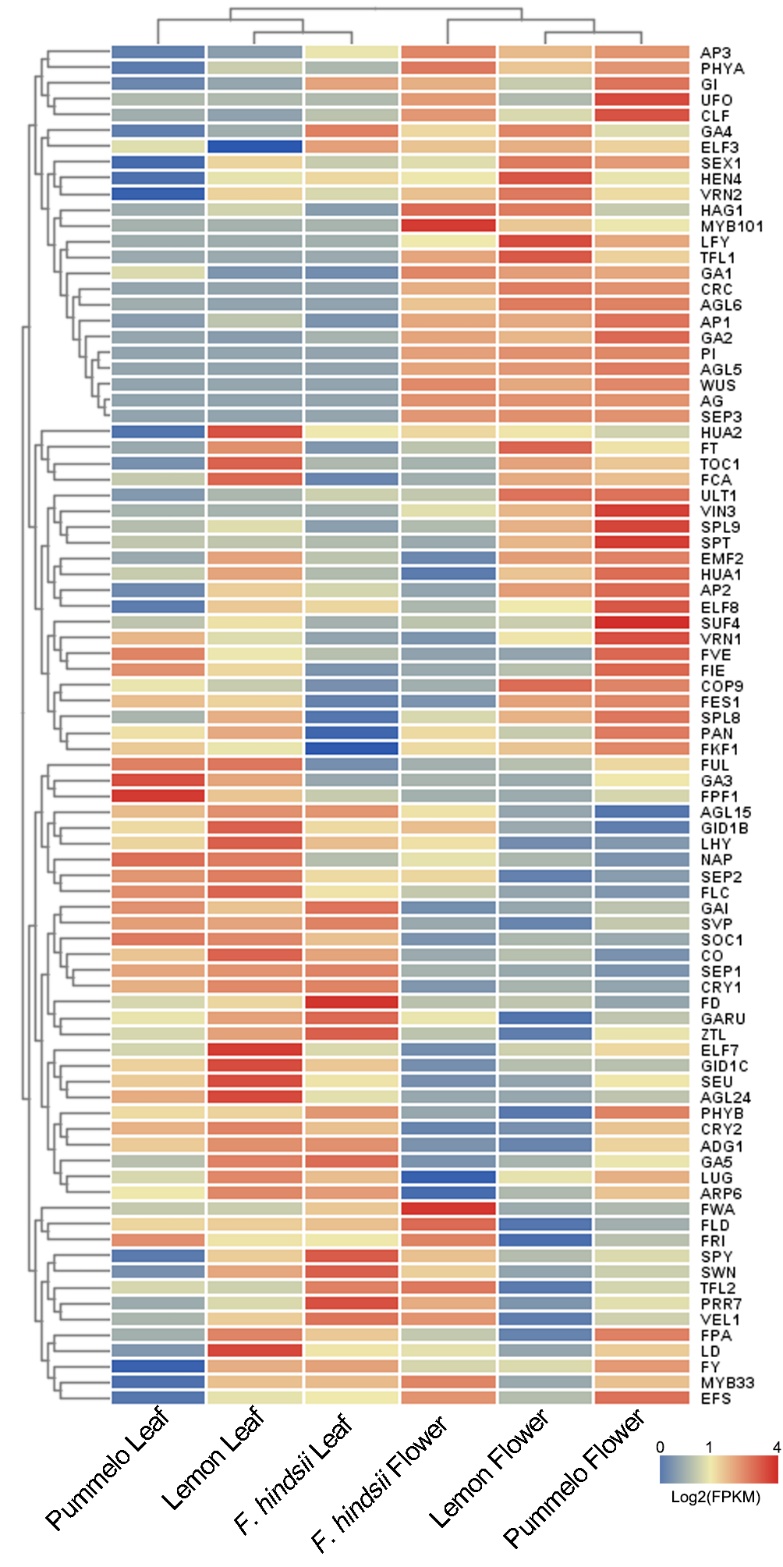


**Figure S7. Expression Pattern of Genes Involved in Flowering in *F. hindsii*, Pummelo and Lemon.**

Heat map of the normalized RNA-Seq data for genes involved in plant flowering.


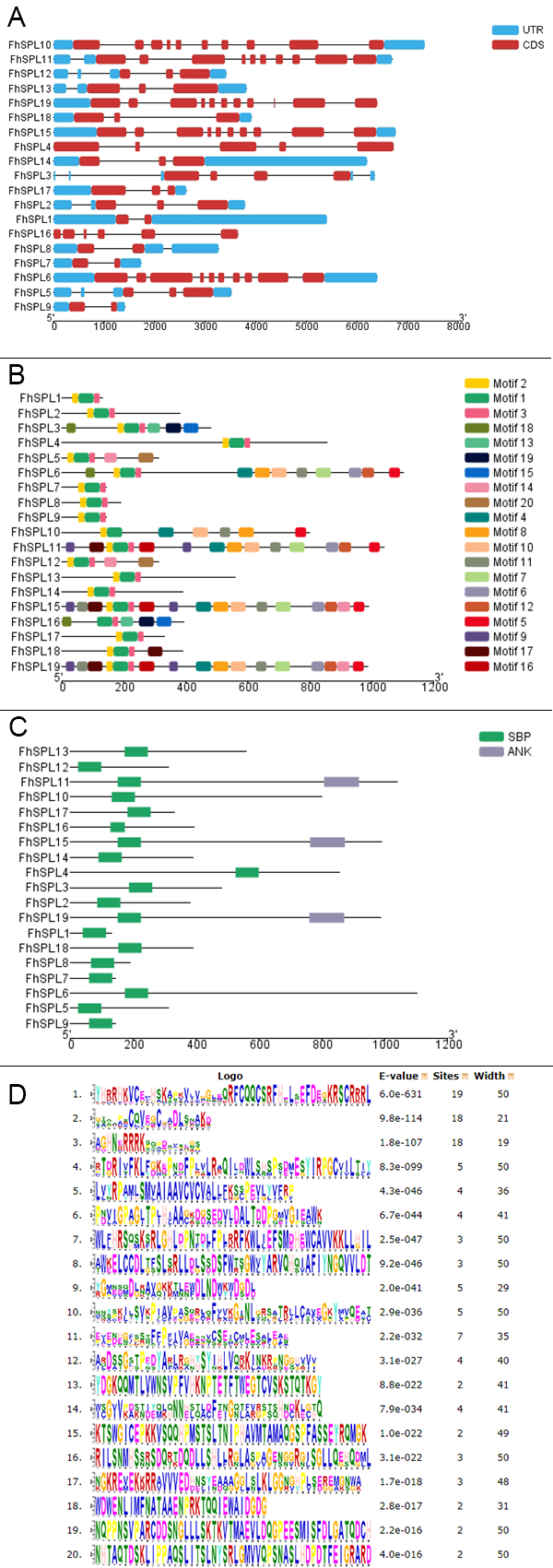


**Figure S8. Genome-wide SPL Identification of *F. hindsii*.**

1. Gene structure of *FhSPLs*. Blue bars indicate untranslated regions (UTR) and Red bars indicate coding sequences (CDS). Fine lines between bars indicate introns.

**(B)** Conserved and potential motifs in FhSPL protein sequences predicted by MEME webtool.

**(C)** Conserved domains in FhSPL protein sequences predicted by NCBI-CCD webtool.

**(D)** Logo sequences of identified motifs in **(B)**. Overall height of each stack represents conservation of the sequence at that position. Each color of the English letters represents a type of amino acid residue.


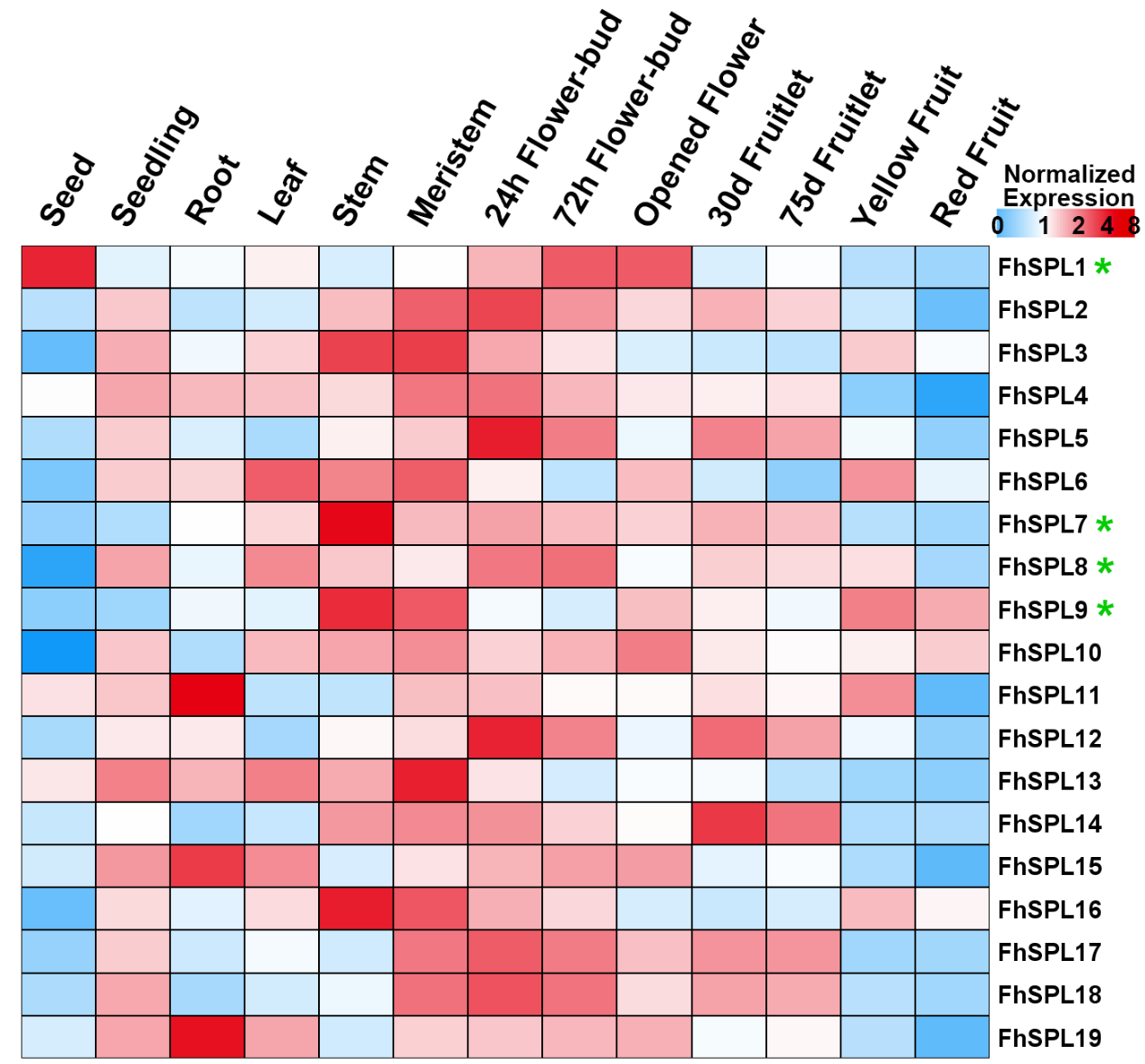


**Figure S9. Expression Pattern of *FhSPLs*.**

Expression levels (Log2 FPKM) of the *FhSPLs* are represented by the color of cell.

Green asterisks indicate the *AtSPL3/4/5* homologous in *F. hindsii* genome.


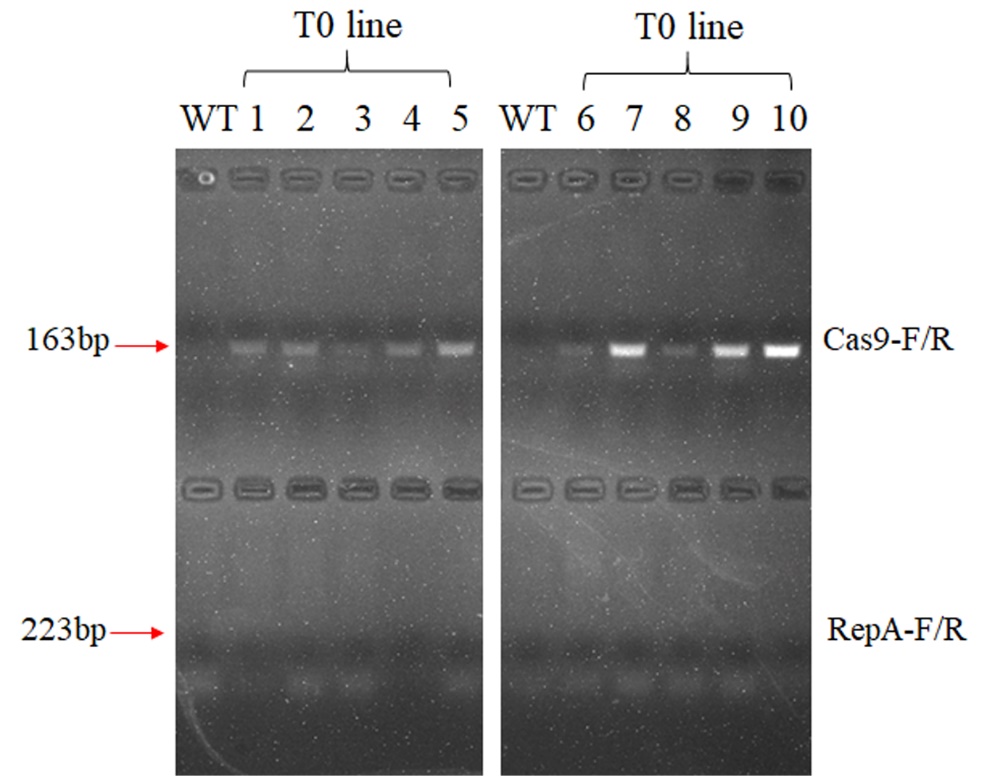


**Figure S10. Positive Identification of Transgenic *F. hindsii* by PCR amplification.**

The positive transgenic plants were identified by primers Cas9-F and Cas9-R (Supplemental Table 15). The size of positive fragment is 163bp. The contamination of agrobacterium residue was checked by primer RepA-F and RepA-R (Supplemental Table 15). No 223 fragment was detected by PCR amplification.


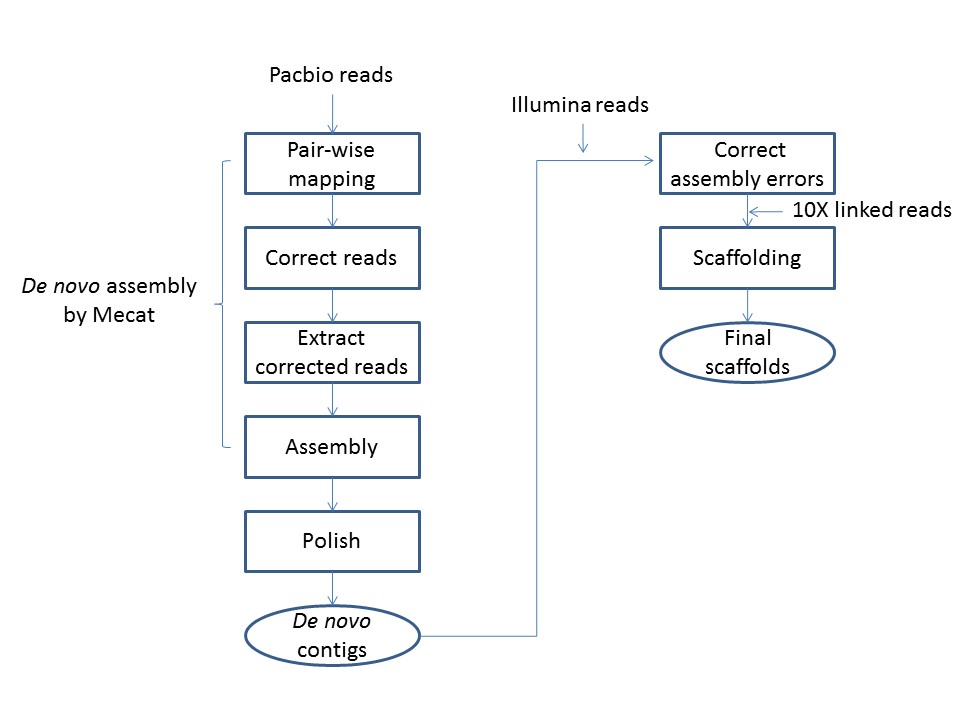


**Figure S11. Brief Workflow of Genome Sequencing and Assembly in Present Study.**


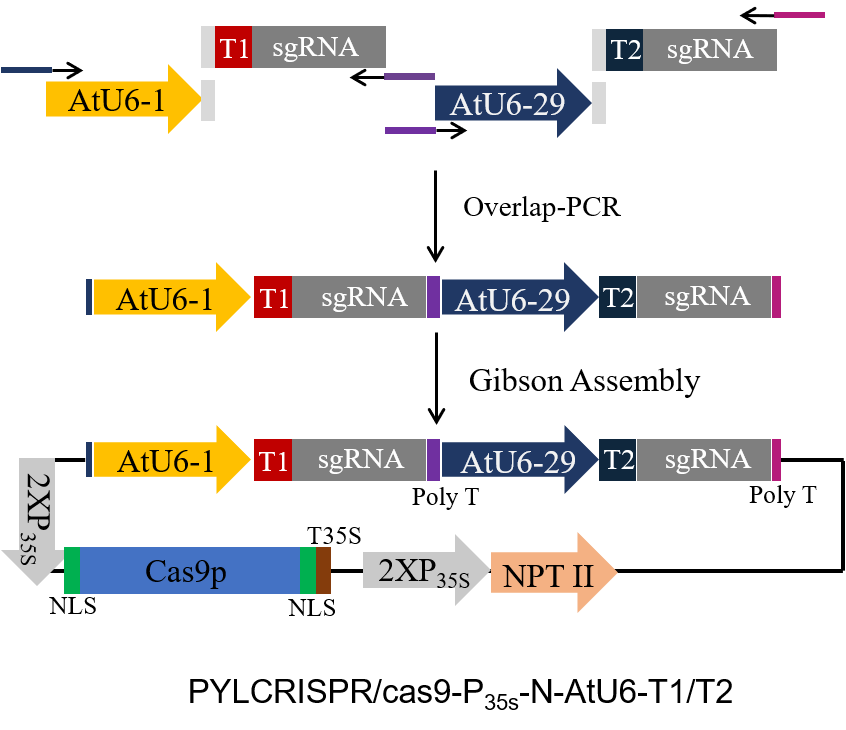


**Figure S12. Diagram of CRISPR/gRNA Vector Construction via Overlap-PCR and Gibson Assembly.**

Illustration of cloning of two sgRNA expression cassettes into the CRISPR/Cas9 binary vector (Ma et al., 2015b) by Gibson Assembly. NPT II, neomycin phosphotransferase II; NLS, nuclear localization sequence; T1, target sequence 1 in target-sgRNA expression cassette 1; T2, target sequence 2 in target-sgRNA expression cassette 2; Poly T, RNA polymerase III termination signal; T35s, CaMV 35S terminator.

| **Province** | **County** | **Sample Number** | **Embryo Feature** |
| --- | --- | --- | --- |
| Fujian | Nanping | 47 | Polyembryony |
|  | Longyan | 210 | Polyembryony/Monoembryony |
|  | Quanzhou | 38 | Polyembryony |
|  | Ningde | 38 | Polyembryony |
|  | Sanming | 65 | Polyembryony |
|  | Xiamen | 15 | Polyembryony |
| Hunan | Chenzhou | 78 | Polyembryony |
|  | Zixing | 9 | Polyembryony |
| Jiangxi | Chongyi | 14 | Polyembryony |
|  | Quannan | 18 | Polyembryony |
|  | Dingnan | 26 | Polyembryony |
|  | Anyuan | 22 | Polyembryony |
|  | Ganzhou | 6 | Polyembryony |
| Zhejiang | Linhai | 16 | Polyembryony |
|  | Ninghai | 32 | Polyembryony |
|  | Taizhou | 42 | Polyembryony |
|  | Cangnan | 34 | Polyembryony |
| Guangdong | Shaoguan | 38 | Polyembryony |
|  | Conghua | 12 | Polyembryony |
|  | Jieyang | 8 | Polyembryony |
|  | Meizhou | 70 | Polyembryony |
| Total |  | 838 |  |

**Table S1. Summary of *F. hindsii* Germplasm Collection.**

**Table S2. Summary of *F. hindsii* Selfing Lines.**

See the supporting material “3. Table S2, S6, S7, S10 and S11.xlsx”.

| **Accession ID** | **Breeding Code** | **K-mer Number** | **K-mer Depth** | **Genome Size (Mbp)** | **Heterozygous Ratio (%)** |
| --- | --- | --- | --- | --- | --- |
| S2s-178 | LS01-01-04 | 2.36E+10 | 62 | 382.9 | 1.07% |
| S2y-56 | SY02-02-30 | 1.57E+10 | 39 | 403.04 | 1.00% |
| S2y-69 | SY02-02-31 | 2.00E+10 | 53 | 378.11 | 0.79% |
| S3f-156 | FC01-02-02-01 | 2.48E+10 | 67 | 367.24 | 0.66% |
| S3t-66 | LT01-01-01-02 | 1.98E+10 | 50 | 393.33 | 0.67% |
| S3y-45 | SY02-02-03-02 | 2.27E+10 | 63 | 358.89 | 0.62% |
| Mean |  |  |  | 380.59 | 0.80% |

**Table S3. Summary of Genome Survey of Outstanding *F. hindsii* Selfed Offspring.**

Data were produced by 17-mer analysis.

| **Sample** | **Mean-X %** | **Genome Size (Mb)** |
| --- | --- | --- |
| *F. hindsii*-1 | 56.38 | 418.77 |
| *F. hindsii*-2 | 56.04 | 409.61 |
| *F. hindsii*-3 | 56.33 | 359.10 |
| *F. hindsii*-4 | 55.11 | 382.40 |
| *F. hindsii*-5 | 56.67 | 421.44 |
| Mean | 56.11 | 398.26 |
| XC-1 (*C. sinensis*) | 49.41 |  |
| XC-2 | 50.21 |  |
| XC-3 | 57.57 |  |
| XC-4 | 52.89 |  |
| XC-5 | 49.35 |  |
| Mean | 51.89 |  |

**Table S4.** **Estimation of *F. hindsii* Genome Size via Flow Cytometry**

*C. sinensis* genome size is 367 Mb.

| **TE Classification** | **Copies (number)** | **Length (bp)** | **Percent of genome** |
| --- | --- | --- | --- |
| LTR/Gyspy | 37,017 | 44,501,054 | 11.91% |
| LTR/Copia | 43,224 | 36,570,103 | 9.79% |
| LTR/Caulimovirus | 8,509 | 10,583,568 | 2.83% |
| LINE | 8,963 | 6,271,814 | 1.68% |
| SINE | 327 | 116,712 | 0.03% |
| DNA/hat | 23,172 | 9,755,916 | 2.61% |
| DNA/EnSpm | 9,176 | 3,302,356 | 0.88% |
| DNA/PIF | 9,379 | 2,780,598 | 0.74% |
| DNA/MuDR | 15,069 | 6,610,118 | 1.77% |
| DNA/Tc1 | 4,072 | 1,104,496 | 0.30% |
| DNA/Helitron | 8,974 | 3,722,011 | 1.0% |
| Simple Repeat | 4,056 | 1,896,885 | 0.51% |
| Satellite | 154 | 30,588 | 0.01% |
| Unknown | 96,073 | 36,588,595 | 9.79% |
| **Total** |  | **163,834,814** | **43.9%** |

**Table S5.** **TE Classification of *F. hindsii* Genome.**

**Table S6. IDs of *F. hindsii* Specific Genes.**

See the supporting material “3. Table S2, S6, S7, S10 and S11.xlsx”.

**Table S7. GO Enrichment Analysis of Genes Specific to *F. hindsii***

See the supporting material “3. Table S2, S6, S7, S10 and S11.xlsx”.

| **Gene Locus** | **Annotation** | **Gene Name** |
| --- | --- | --- |
| sjg198540 | Floral Induction GA | GA1 |
| sjg289620 | Floral Induction GA | GA2 |
| **sjg088850** | Floral Induction GA | GA3 |
| sjg014520 | Floral Induction GA | GA4 |
| **sjg288790** | Floral Induction GA | GA5 |
| sjg070650 | Floral Induction GA | SPY |
| sjg087150 | Floral Induction GA | FPF1 |
| sjg137020 | Floral Induction GA | GID1B |
| sjg040340 | Floral Induction GA | GID1C |
| **sjg075680** | Floral Induction GA | GARU |
| sjg277850 | Floral Induction GA | GAI |
| sjg113320 | Floral Induction Photoperiod | CO |
| sjg107000 | Floral Induction Photoperiod | COP9 |
| sjg062570 | Floral Induction Photoperiod | CRY1 |
| sjg130340 | Floral Induction Photoperiod | CRY2 |
| **sjg280110** | Floral Induction Photoperiod | FKF1 |
| sjg088480 | Floral Induction Photoperiod | ELF3 |
| **sjg160910** | Floral Induction Photoperiod | FT |
| sjg063200 | Floral Induction Photoperiod | GI |
| **sjg259520** | Floral Induction Photoperiod | LHY |
| **sjg261520** | Floral Induction Photoperiod | PHYA |
| sjg135890 | Floral Induction Photoperiod | PHYB |
| **sjg170730** | Floral Induction Photoperiod | PRR7 |
| **sjg294430** | Floral Induction Photoperiod | SOC1 |
| **sjg186850** | Floral Induction Photoperiod | TOC1 |
| sjg020780 | Floral Induction Photoperiod | ZTL |
| **sjg037990** | Floral Induction Photoperiod | FD |
| sjg302510 | Floral Induction Photoperiod | ELF7 |
| **sjg296810** | Floral Induction Photoperiod | AGL6 |
| **sjg003840** | Floral Induction Photoperiod | TFL2 |
| sjg262200 | Floral Induction Photoperiod | AGL24 |
| sjg151670 | Floral Induction Vernalization | ARP6 |
| **sjg130820** | Floral Induction Vernalization | FCA |
| **sjg026670** | Floral Induction Vernalization | FLC |
| **sjg139610** | Floral Induction Vernalization | FLD |
| sjg114920 | Floral Induction Vernalization | FPA |
| sjg213920 | Floral Induction Vernalization | FRI |
| sjg283210 | Floral Induction Vernalization | FVE |
| **sjg287260** | Floral Induction Vernalization | FWA |
| sjg176580 | Floral Induction Vernalization | LD |
| sjg316490 | Floral Induction Vernalization | SVP |
| sjg145140 | Floral Induction Vernalization | VRN1 |
| sjg264760 | Floral Induction Vernalization | VRN2 |
| sjg013280 | Floral Induction Vernalization | FIE |
| sjg019520 | Floral Induction Vernalization | VEL1 |
| sjg163610 | Floral Induction Vernalization | FY |
| sjg276530 | Floral Induction Vernalization | SUF4 |
| sjg122470 | Floral Induction Vernalization | FES1 |
| sjg219380 | Floral Induction Vernalization | ELF8 |
| sjg064520 | Floral Induction Vernalization | EFS |
| sjg177130 | Floral Induction Vernalization | SWN |
| **sjg182870** | Floral Induction Vernalization | VIN3 |
| sjg122830 | Floral Determination | AG |
| **sjg029130** | Floral Determination | AP1 |
| **sjg321180** | Floral Determination | AP2 |
| **sjg038400** | Floral Determination | AP3 |
| **sjg187680** | Floral Determination | FUL |
| sjg214280 | Floral Determination | LFY |
| sjg151600 | Floral Determination | LUG |
| sjg069570 | Floral Determination | TFL1 |
| sjg280720 | Floral Determination | UFO |
| sjg319510 | Floral Determination | WUS |
| **sjg108690** | Floral Determination | PAN |
| sjg101620 | Floral Determination | ULT1 |
| sjg061860 | Floral Determination | SEU |
| **sjg009510** | Floral Determination | AGL15 |
| **sjg210450** | Floral Determination | SPL9 |
| sjg195380 | Floral Determination | HAG1 |
| sjg192270 | Floral Determination | EMF2 |
| sjg064160 | Floral Determination | PI |
| sjg317470 | Floral Morphogenesis | CLF |
| sjg028880 | Floral Morphogenesis | CRC |
| sjg287850 | Floral Morphogenesis | HUA1 |
| sjg108350 | Floral Morphogenesis | HUA2 |
| sjg256830 | Floral Morphogenesis | HEN4 |
| **sjg008020** | Floral Morphogenesis | NAP |
| sjg146680 | Floral Morphogenesis | SEP1 |
| **sjg052220** | Floral Morphogenesis | SEP2 |
| sjg028010 | Floral Morphogenesis | SEP3 |
| sjg054740 | Floral Morphogenesis | AGL5 |
| **sjg314170** | Floral Morphogenesis | SPT |
| **sjg294320** | Floral Morphogenesis | SPL8 |
| sjg281800 | Other | ADG1 |
| **sjg106360** | Other | MYB33 |
| **sjg291100** | Other | MYB101 |
| sjg006500 | Other | SEX1 |

**Table S8. The Flowering Gene Selected for Expression Analysis.**

Bold-type marks the different expression genes between *F. hindsii* and pummelo/lemon.

| **Locus ID** | **Gene/Protein Name** |
| --- | --- |
| sjg000230 | FhSPL1 |
| sjg297880 | FhSPL10 |
| sjg017640 | FhSPL11 |
| sjg189650 | FhSPL12 |
| sjg026850 | FhSPL13 |
| sjg134860 | FhSPL14 |
| sjg028180 | FhSPL15 |
| sjg130440 | FhSPL16 |
| sjg259700 | FhSPL17 |
| sjg262710 | FhSPL18 |
| sjg034920 | FhSPL19 |
| sjg210450 | FhSPL2 |
| sjg120290 | FhSPL3 |
| sjg027360 | FhSPL4 |
| sjg174780 | FhSPL5 |
| sjg101840 | FhSPL6 |
| sjg294320 | FhSPL7 |
| sjg295160 | FhSPL8 |
| sjg049180 | FhSPL9 |

**Table S9. Gene IDs of 19 Identified *FhSPLs*.**

**Table S10. Predicted miRNA Target-side of Candidate *FhSPLs***

See the supporting material “3. Table S2, S6, S7, S10 and S11.xlsx”.

**Table S11. Rate of CRSIPR/gRNA Modified Various Nucleotide Insertion (+) and Deletion (-) Events in *F. hindsii* Transgenic Lines**

See the supporting material “3. Table S2, S6, S7, S10 and S11.xlsx”.

| **Genes** | **Transgenic Lines** | **Putative Off-target Genes** | | |  | **Off-targets Lines** | | |  |
| --- | --- | --- | --- | --- | --- | --- | --- | --- | --- |
|  |  | gRNA1^a^ |  | gRNA2^b^ |  | gRNA1 |  | gRNA2 | |
| CCD4b | T0-#70 | AACCCACAACACATGCCACG**TGG** (Cs8g14180)  AACCCACAGTACCTCCCTCG**CGG** (Cs7g14820) |  | CAACCACTGGGCACTCGGTG**GGG** (Cs8g14180)    CAATCTCCGGGCACTCACTC**CAG** (Cs2g03630)  CACCCACTGGCCACTCATTC**TGG** (Cs9g07990) |  | 0 |  | 0 | |
|  | T0-#40 |  |  |  |  | 0 |  | 0 | |
|  | T1-#40-1 |  |  |  |  | 0 |  | 0 | |
|  | T1-#40- 2 |  |  |  |  | 0 |  | 0 | |
|  | T1-#40- 3 |  |  |  |  | 0 |  | 0 | |
|  | T1-#40- 4 |  |  |  |  | 0 |  | 0 | |
|  | T1-#40- 5 |  |  |  |  | 0 |  | 0 | |
|  | T1-#40- 6 |  |  |  |  | 0 |  | 0 | |
| PDS | T0-#1 | GGACAGCCACTCCTCAGCCG**AGG** (Cs1g24480) |  | TCTGCAGCAGACTTTTGTAT**CGG** (Cs2g28920) TCTTCAGCAAACCTTTTTAA**TGG** (Cs7g28840) |  | 0 |  | 0 | |
|  | T0-#3 |  |  |  |  | 0 |  | 0 | |
|  | T0-#6 |  |  |  |  | 0 |  | 0 | |
|  | T0-#9 |  |  |  |  | 0 |  | 0 | |
|  | T0-#10 |  |  |  |  | 0 |  | 0 | |

**Table S12.** **Summary of Putative Off-target Analysis of CRISPR/Cas9-trasgenic *F. hindsii*.**

| **Code** | **Original code** | **Motif** | **Forward primer** | **Reversed primer** | **Chromosome** |
| --- | --- | --- | --- | --- | --- |
| M1-2 | M1H4Si8076 | (TTTA)5 | GATCATCTTGTGCGGAACAC | AGGAGTTGGGGATTGTGAAA | Chr1 |
| M1-5 | M1H5Si17929 | (ATGCC)4 | TGACTGGTGAAACGGTTTGT | AACATCGTTACTCGCTGCAC | Chr1 |
| M1-19 | M1H2Si16887 | (AG)8 | GTCATGAAACCAGCACCATC | GGCATGTCATGGTTATTTGG | Chr1 |
| M2-3 | M2H3Si3169 | (AAT)6 | CTTCACCGTAACATCAACGG | GTGGATGCAATGCAGAAGTT | Chr2 |
| M2-13 | M2H2Si20644 | (GT)18 | CGAGAGGAATCATGGGATCT | TGTGTTCAGTGTGGAGTTGC | Chr2 |
| M2-14 | M2H3Si15180 | (GAA)6 | CATAACGCCACCATTAAACG | GAGGCATCTTCTTCAAAGCC | Chr2 |
| M3-2 | M3H3Si763 | (AAG)6 | CGCACTTGAAACGCCTAATA | TCTGCATCACAGTCGTAGCA | Chr3 |
| M3-7 | M3H3Si28170 | (TTC)7 | AAAACTCAATGGCATCCCTC | TTGGTCAGGCTTTTAACACG | Chr3 |
| M3-11 | M3H2Si9115 | (TA)10 | ATTCTTGACCCATTTACCCG | CCCGACACGGAAAAATAATC | Chr3 |
| M4-3 | M4H2Si13358 | (GA)13 | GCATAAATGGAGAGGCCAAT | CACAGGTCAGAGCTGCATTT | Chr4 |
| M4-5 | M4H2Si35917 | (GT)11 | TTGTAGCCCATGTAGCTTGC | TTGTGTGGCATGGATCTCTT | Chr4 |
| M4-10 | M4H2Si10740 | (GA)8 | GCAAGTGAAATTGGCTGAGA | GTGCAATGCGTATTCCATCT | Chr4 |
| M5-4 | M5H2Si22590 | (TC)12 | GCAGATTTGGCAGATTGAGA | CATACACCCCATTCAACAGC | Chr5 |
| M5-8 | M5H10Si4489 | (GAAAGGATGG)3 | CTTTAAGTGTGGACCGCTCA | GGAACCCAAATGAAATGGTC | Chr5 |
| M5-10 | M5H2Si6855 | (AG)9 | GGCAAATAAAACGGACCTTC | AGACGCACAAGTCAAAGAGC | Chr5 |
| M6-3 | M6H4Si23426 | (GAAA)5 | TGTACTCTCGCATTGCCATT | GCTTCTCCAGAAACCATTCC | Chr6 |
| M6-7 | M6H3Si12731 | (ATA)9 | TAGGCTCTTCCTTCCACGTT | AACGCAACACCACTCTGAAG | Chr6 |
| M6-9 | M6H3Si18545 | (ATA)10 | GTGCTTTATTCAAGCCCACG | TTAAAGCACGGCACGATTAG | Chr6 |
| M7-7 | M7H3Si23837 | (ATG)9 | ACCATCCATATTGCAGCGTA | TTGAATACGTCCACCCTTCA | Chr7 |
| M7-10 | M7H2Si19820 | (AC)11 | TACCCACCAAGCGATTCATA | TTGCAAATGACTTCAGCTCC | Chr7 |
| M7-11 | M7H2Si31270 | (AT)12 | GCACTTAGCCATTAATCCGA | GACTTTTGTTATTCCACCCC | Chr7 |
| M8-3 | M8H5Si36426 | (TTCGG)5 | CCATAAATATGTGCGAACCG | TAGGGGCCTCAAATAGGTTG | Chr8 |
| M8-9 | M8H4Si9690 | (TATG)5 | TTGGCAGAAAACAGAGTTGC | CAATGGGAGTCGTTGATTTG | Chr8 |
| M8-10 | M8H3Si9989 | (TAT)6 | TTTGGTCAGCAGATGACTCC | CAGATTGCATTGGTTGTTCC | Chr8 |
| M9-6 | M9H2Si41459 | (GA)8 | GGGGAGGATTCAAGGAATTT | GCCCAAGAGATCGTAGAAGC | Chr9 |
| M9-8 | M9H2Si25847 | (TA)8 | AAAATCTGGATCTGGGTTCG | GCCTGATTTACGGAGGTGAT | Chr9 |
| M9-9 | M9H3Si25423 | (AAT)7 | AGAGACCGTTCCAAAACCTC | GCTGTTGCTGGTTCCTTGTA | Chr9 |

**Table S13. The SSR Markers Used for Homozygosity Estimation.**

These primers and their location information were derived from the sweet orange genome project.

| **Primer Name** | **Sequence (5' to 3')** |
| --- | --- |
| FUL_RT_NEW_F | GGTATTGTTATGCGGAGAGGC |
| FUL_RT_NEW_R | TGCAAGATCCTCTCCCATGA |
| SOC1_RT_NEW_F | GGGAGAAGGTCTGGCATCAT |
| SOC1_RT_NEW_R | TTTGAGCTGCGCAATCTGTT |
| AP1_RT_NEW_F | TTTTGGAGGGGCTTTGGTTG |
| AP1_RT_NEW_R | GCTGAACCCTACCTCTTCCC |
| FD_RT_NEW_F | CCCCTCTGGCTCATCATCAT |
| FD_RT_NEW_R | AGGACACAACATGAGCAGGA |
| FT_RT_NEW_F | ACGATGGGGATTCACAGGTT |
| FT_RT_NEW_R | TCTCCCTCTGGCAGTTGAAG |
| LFY_RT_NEW_F | AGAATGTTGGGGCATGGAGA |
| LFY_RT_NEW_R | CAAGACGGGGATGAGCATTG |
| AGL24_RT_NEW_F | CACAGTGTGCATGCTAGAGAG |
| AGL24_RT_NEW_R | ACTCCAACCTCAGCATCACA |
| FhSPL8_RT_NEW_F | GCGGCTTTTGTGGATTCAGA |
| FhSPL8_RT_NEW_R | CTCCTGTGGTACTGCTTTGC |
| FhSPL1_RT_NEW_F | TCCAAGGCTCCTGTTGTGAT |
| FhSPL1_RT_NEW_R | TCATTGTGTCCAGCCAAACG |
| FhSPL7_RT_NEW_F | TGGTGGTATTGCTGGAGAGG |
| FhSPL7_RT_NEW_R | CACGAACAACAGGAGCCTTG |
| FhSPL9_RT_NEW_F | TGGTGGTATTGCTGGAGAGG |
| FhSPL9_RT_NEW_R | CACGAACAACAGGAGCCTTG |
| FhSPL14_RT_NEW_F | CAGTGTTCAACAGCTGGGAC |
| FhSPL14_RT_NEW_R | GTGACGGATTTGGTATGGGC |
| miR156aNEW-QRT-F | TCGTATCCAGTGCAGGGTCCGA |
| miR156aNEW-QRT-R | GCGGCGGTTGACAGAAGAGAGT |
| miR172bNEW-QRT-F | TCGTATCCAGTGCAGGGTCCGA |
| miR172bNEW-QRT-R | CGGCGGCGAGAATCTTGATGATG |
| loop-miR156a | GTCGTATCCAGTGCAGGGTCCGAGGTATTCGCACTGGATACGACGTGCTC |
| loop-miR172b | GTCGTATCCAGTGCAGGGTCCGAGGTATTCGCACTGGATACGACATGCAG |

**Table S14. Primer Used for qRT-PCR Experiment.**

| Primer ID | Primer sequence (5′→3′) |
| --- | --- |
| CRISPR-1 | ACCGGTAAGGCGCGCCGTAGTGCTCGATGGAATCGGCAGCAAAGG |
| CRISPR-2 | CTCCGTTTTACCTGTGGAATCG |
| CRISPR-3 | TAGCTCGAGAGGCGCGCCAATGATACCGCCATCCACTCCAAGCTCT |
| CRISPR-4 | GCTTGGAGTGGATGGCTCCCATGGGAACAACAG |
| CRISPR-5 | TTGCTGCCGATTCCACCATCCACTCCAAGCTCT |
| CRISPR-6 | CGGAGGAAAATTCCATCCAC |
| CRISPR-7 | ACCGGTAAGGCGCGCCGTA |
| CRISPR-8 | TAGCTCGAGAGGCGCGCCAA |
| CRISPR-PDS-1 | AAGGTCTCCTGTAGA CAATCACTACTTCGTCTC |
| CRISPR-PDS-2 | GAAGAGTGGATCTCA CAATCTCTTAGTCGACTC |
| CRISPR-PDS-3 | ACGAAGTAGTGATTG TCTACAGGAGACCTTTGTAA |
| CRISPR-PDS-4 | TCGACTAAGAGATTGTGAGATCCACTCTTCAGCCG |
| CRISPR-PDS-5 | TACAGGAGACCTTTGTAAGTTTTAGAGCTAGAAATA |
| CRISPR-PDS-6 | AGATCCACTCTTCAGCCGGTTTTAGAGCTAGAAATA |
| CRISPR-CCD4b-1 | AGTGCCCGGTGGTTGCAATCACTACTTCGTCTC |
| CRISPR-CCD4b-2 | CATGTGCTGTGGGTTCAATCTCTTAGTCGACTC |
| CRISPR-CCD4b-3 | ACGAAGTAGTGATT GCAACCACCGGGCACTCAGTC |
| CRISPR-CCD4b-4 | TCGACTAAGAGATTGAACCCACAGCACATGCCCCG |
| CRISPR-CCD4b-5 | ACCACCGGGCACTCAGTCGTTTTAGAGCTAGAAATA |
| CRISPR-CCD4b-6 | CCCACAGCACATGCCCCGGTTTTAGAGCTAGAAATA |
| Cas9-F | GATCTCGCGGACCTTGTAGA |
| Cas9-R | GAGGCAGATTACCAAGCACG |
| RepA-F | CGGAAAGCAGAAAGACGACC |
| RepA-R | CTTGCCTTCTGTGATCTCGC |
| PDS-detect-F | ATGAGACGCCAAAGTGAGGA |
| PDS-detect-R | CCCTCCCTCCATGAAAAGGT |
| CCD4b-detect-F | CCGCCAAAACCAAAGTTCACA |
| CCD4b-detect-R | TTCCTTGGCACAAATAATGGC |
| HRM-CCD4b-left-F | AATCCGCGCCATCATTGTTT |
| HRM-CCD4b-left-R | ACATGCCGGTTAGAGAGTCC |
| HRM-CCD4b-right-F | GGACTCTCTAACCGGCATGT |
| HRM-CCD4b-right-R | ATCCTGAGAAGACGTTGGGG |
| PDS-Cs2g28920-F | CGCAGCACTTCTTGGATA |
| PDS-Cs2g28920-R | CAGGAATCACTGGCATCT |
| PDS-Cs7g28840-F | TACCCGCAGATTTATCGC |
| PDS-Cs7g28840-R | TACCCTCAAGTTTGTTAGCATC |
| PDS-Cs1g24480-F | CGCCGTGACATTTAGGTT |
| PDS-Cs1g24480-R | TTGTTCAAGGGCTTTAGG |
| 4b-Cs8g14180-F | GTGTTCCCATACGGTCTTCAA |
| 4b-Cs8g14180-R | AATCCCATCTCCCTACAGTTT |
| 4b-Cs2g03630-F | CGCAGAAGACAAGCACATAGC |
| 4b-Cs2g03630-R | TTCGTTTCACTTTCATCCGTCCCA |
| 4b-Cs9g07990-F | AGTTGACCTACTTTGCCTTGT |
| 4b-Cs9g07990-R | TGTCTTCCAACCCAGTAGCGT |
| 4b-Cs7g14820-F | CCATAAATCCACGAAGCCAACT |
| 4b-Cs7g14820-R | GTCTACGAACACCGCATACTTT |

**Table S15. Primers Used in CRISPR Experiment.**

**Note:** CRISPR-1–8 and CRISPR-PDS-1–6 were used for cloning of two sgRNA expression cassettes of PDS gene in the pYLCRISPR/Cas9P_35S_-N Vectors by using overlapping PCR and Gibson assembly method (Ma et al., 2015). CRISPR-1~8 and CRISPR-CCD4b-1~6 primers were used for CRISPR/Cas9 vector construction of CCD4b gene. Cas9-F and Cas9-R were used for identification of positive transgenic plants. RepA-F and RepA-R were used to check contamination of agrobacterium residue. PDS-detect-F&R and CCD4b-detect-F&R were used to test the editing results of mutagenesis of PDS gene and CCD4b gene respectively. HRM-CCD4b-left-F&R and HRM-CCD4b-right-F&R were used for HRM analysis. PDS-Cs2g28920-F&R, PDS-Cs7g28840-F&R and PDS-Cs1g24480-F&R were used for off-target analysis in CRISPR plant with mutagenesis of PDS gene. 4b-Cs8g14180-F&R, 4b-Cs2g03630-F&R, 4b-Cs9g07990-F&R and 4b-Cs7g14820-F&R were used for off-target analysis in *ccd4b* mutant.
